# Photochemical modifications for DNA/RNA oligonucleotides

**DOI:** 10.1039/d1ra05951c

**Published:** 2022-02-24

**Authors:** Amirrasoul Tavakoli, Jung-Hyun Min

**Affiliations:** Department of Chemistry & Biochemistry, Baylor University Waco TX 76706 USA JungHyun_Min@baylor.edu +1-254-710-2095

## Abstract

Light-triggered chemical reactions can provide excellent tools to investigate the fundamental mechanisms important in biology. Light is easily applicable and orthogonal to most cellular events, and its dose and locality can be controlled in tissues and cells. Light-induced conversion of photochemical groups installed on small molecules, proteins, and oligonucleotides can alter their functional states and thus the ensuing biological events. Recently, photochemical control of DNA/RNA structure and function has garnered attention thanks to the rapidly expanding photochemistry used in diverse biological applications. Photoconvertible groups can be incorporated in the backbone, ribose, and nucleobase of an oligonucleotide to undergo various irreversible and reversible light-induced reactions such as cleavage, crosslinking, isomerization, and intramolecular cyclization reactions. In this review, we gather a list of photoconvertible groups used in oligonucleotides and summarize their reaction characteristics, impacts on DNA/RNA thermal stability and structure, as well as their biological applications.

## Abstract 2

Photoconvertible groups (PG) can be incorporated into an oligonucleotide to undergo various irreversible and reversible light-induced reactions such as cleavage, crosslinking, isomerization, and intramolecular cyclization reactions.

## Graphical abstract

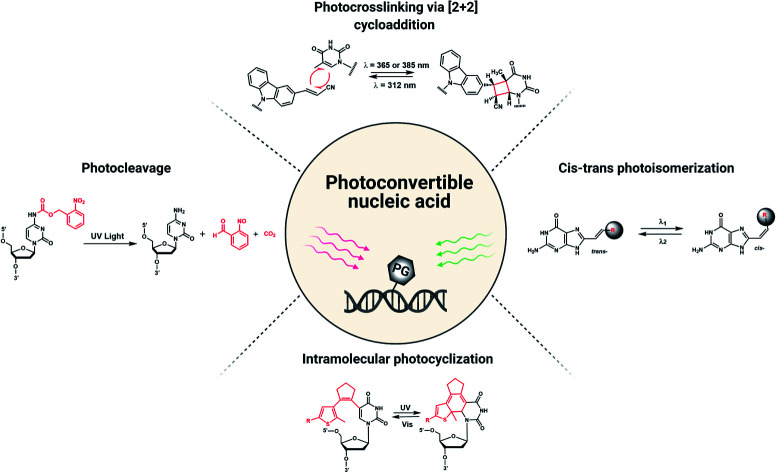

## Introduction

Optical control of chemical reactions has recently gained popularity.^1–4^ These controls rely on photoconvertible groups that undergo structural changes upon irradiation by light.^5–10^ Light can be readily applied to and removed from a reaction, and the wavelength, localization, and intensity of the light can be precisely controlled.^11,12^ Thus, light offers distinct advantages in triggering and controlling reactions, compared with other more common methods such as chemical inhibition, rapid mixing, temperature-, salt- or pH-jumps. In some reversible reactions, the forward or reverse reactions are promoted by distinct wavelengths of light, offering a unique advantage.^13,14^

Photoconvertible modifications in small molecules,^15,16^ oligonucleotides,^12,17,18^ peptides,^19^ and proteins (mostly enzymes)^20–24^ have been applied to control and monitor biological events such as gene expression, enzyme activity, oligomerization states, cellular localization, and immune responses. Here, we have compiled a list of photoreactive modifications on DNA/RNA oligonucleotides and summarized the literature on their reaction characteristics, impacts on DNA/RNA thermal stability and structure, as well as their biological applications (Table 1).

Many photochemical groups entail ‘bulky’ modifications that alter the DNA/RNA structures in unique ways and some modifications can induce site-specific strand-breaks, thus mimicking cellular DNA damage. Thus, these applications may be applicable to studying various DNA damage repair and response mechanisms as well as in the more commonly used applications such as gene expression control. We hope this review will provide useful information for the community of researchers looking for ways to use light to study biochemical/molecular events.

## Photochemical modifications for DNA/RNA oligonucleotides

Photochemical modifications are most commonly incorporated into oligonucleotides by solid-phase synthesis using phosphoramidite chemistry in which a phosphoramidite building block containing the desired photochemical group is first synthesized and subsequently incorporated to an oligonucleotide chain.^25^ Post-synthetic approaches have also been used in which site-specific chemical reactions were carried out directly on nucleic acids: this approach can bypass the need for specialized equipment such as DNA/RNA synthesizer.^26–28^ Most of the modifications in this review are incorporated into oligonucleotides *via* the phosphoramidite chemistry unless noted otherwise.

The family of photochemical groups for oligonucleotides (1 to 12) is named after the parent molecule and can be grouped into four broad categories (I to IV) according to their reaction types as follows:

**I. Photocleavage − irreversible:**

(1) *o*-Nitrobenzyl

(2) *p*-Hydroxyphenacyl

(3) TEEP-OH

(4) Aryl sulfide

(5) Nitroindole

(6) Benzophenone/acetophenone

(7) Coumarin

**II. Intermolecular photocrosslinking *via* [2 + 2] cycloaddition − reversible**

(7) Coumarin

(8) Carbazole

(9) Vinyl derivatives

**III. *Cis–trans* photoisomerization − reversible**

(9) Vinyl derivative

(10) Azobenzene

**IV. Intramolecular photocyclization − reversible**

(11) Spiropyrans

(12) Diarylethene

### 1. *o*-Nitrobenzyl

**Fig. 1:**
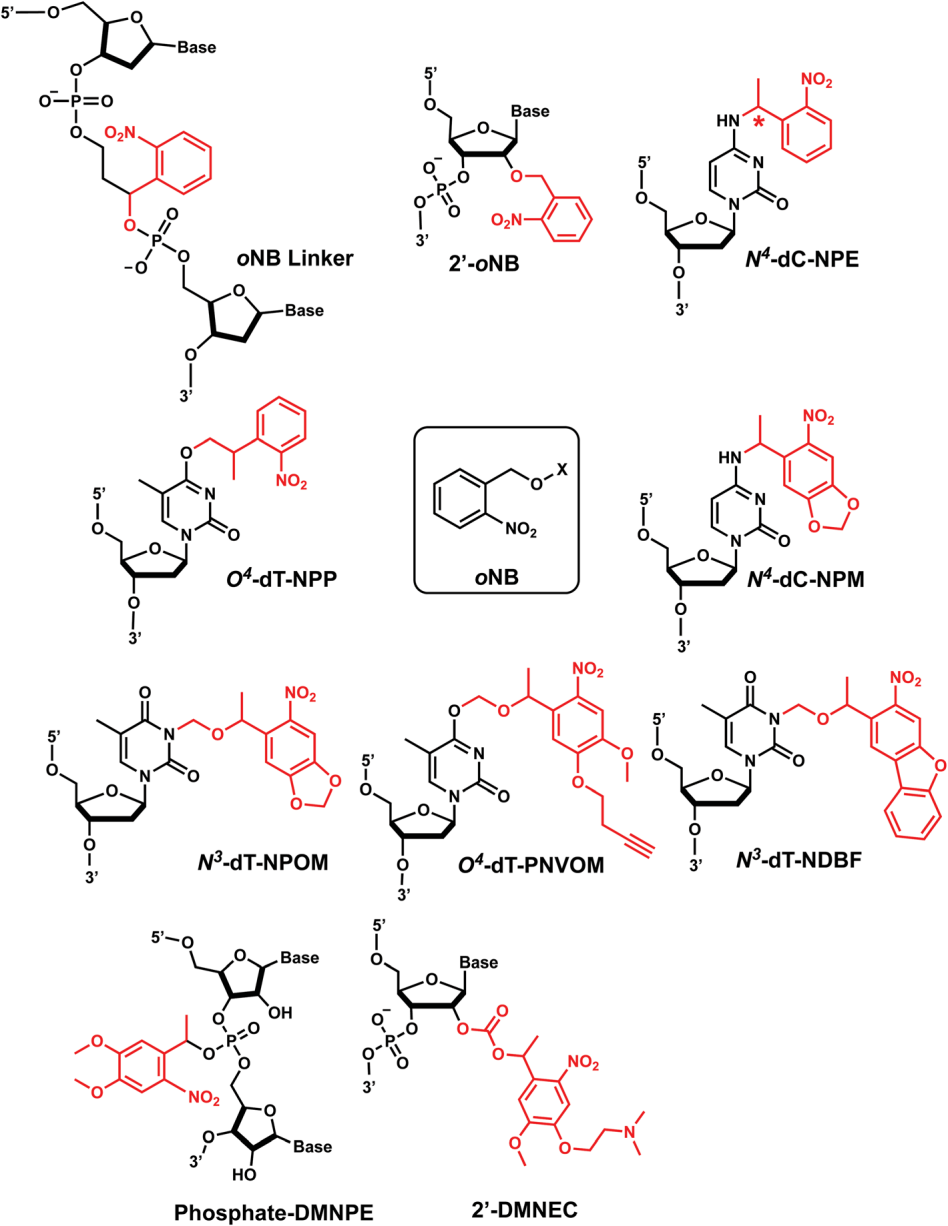
Representative structures of *o*NB modifications on oligonucleotides. *o*NB's X represents a caged substrate such as an oligonucleotide. Other types of *o*NB derivatives are also available but not shown for clarity.

*ortho*-Nitrobenzyl (*o*NB) group is the most extensively studied and applied photoremovable group. A wide variety of functional groups can be introduced into the *o*NB scaffold, and *o*NB derivatives have been used as a part of DNA, RNA, small molecules, and proteins.^11^ Photocleavage wavelengths are tunable (*λ* = 345–420 nm).^29^ In DNA/RNA, they can be incorporated in the backbone, ribose, or nucleobase. Initially, *o*NB derivatives have been employed as a part of the backbone linkers that can trigger light-induced strand breaks (Fig. 1).^30–32^ These cleavable *o*NB linkers have been used in various systems including circular antisense oligonucleotides,^33^ DNAzyme,^34^ negatively charged peptide nucleic acids,^35^ single-stranded circular RNAs as RNA interference (siRNA) precursors,^36^ single guide RNA (sgRNA) for CRISPR-Cas9-based gene editing,^37–39^ and splice-switching oligonucleotides.^40^ Caging the 2′-OH with *o*NB group^41,42^ has been applied to regulate DNAzymes by the Lu group (Fig. 1).^43–46^

*o*NB and its derivatives have also been incorporated in nucleobases to make caged nucleobases for various applications (reviewed by Deiters^30^). 1-(*ortho*-Nitrophenyl)-ethyl (NPE) and 2-(*ortho*-nitrophenyl)-propyl (NPP) caged nucleotides were among the first *o*NB-modified nucleobase.^30^ Later, 6-nitropiperonyl methyl group (NPM) on N^4^-dC and its corresponding hydroxymethylene analogs (NPOM) on N^3^-dT, N^3^–U and N^1^-dG were developed; these groups offered longer photocleavage wavelengths (∼365 nm) than that used for *o*NB and better stability in an aqueous environment at various pHs.^30^ In particular, NPOM, developed by the Deiters group, has been extensively used for various *in vitro* and *in vivo* biological applications.^9,40,47–51^

Closely related propargyl-6-nitroveratryloxymethyl (PNVOM) modification contains an alkyne group available for post-synthetic click reaction.^52^ The nitrodibenzofuran (NDBF) group attached to N^3^-dT^53^ or N^4^-dC and N^6^-dA^54^ showed photocleavage with >400 nm wavelength.^54^ 4,5-Dimethoxy-2-nitrophenylethyl (DMNPE) has been used to modify internal^55^ and termini^56^ phosphate in siRNA. 1-(4-(2-(Dimethylamino)ethoxy)-5-methoxy-2-nitrophenyl)ethyl carbonyl (DMNEC) moiety has been utilized for acylating 2′-hydroxyls of RNA.^26,57^

#### Reaction characteristics

**Fig. 2:**
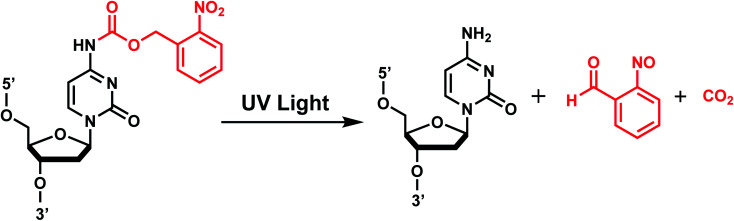
*o*NB photocleavage reaction. Upon irradiation with UV-A light, the bond between the *o*NB and the leaving group (*e.g.*, dC nucleoside) is cleaved in a radical-mediated reaction. The byproduct (*e.g.*, CO_2_) varies depending on the type of *o*NB photocage.

In *o*NB and its derivatives, the caged substrate such as oligonucleotide (X in Fig. 1 center) can be attached to the benzylic ring as a leaving group to be released upon irradiation *via* Norrish type II mechanism, mediated by radicals (Fig. 2; extensively reviewed in ref. 11). Substitutions on the benzylic ring affect the stability as well as the absorption spectra of the caged molecules.^29^ An electron-withdrawing group at the *para*-position to the nitro group of *o*NB or a moderately electron-donating group in the *meta*-position results in a red-shift in the absorption and licenses cleavage with longer wavelengths of light (reviewed in ref. 29).

*o*NB derivatives in oligonucleotides are shown to be removed in seconds to minutes range using a wide range of power mW–W.^39,49,58,59^ In a study by Stephanopoulos *et al.*, 85% removal of NPOM-caged DNA occurred in 3 s using 18.2 W lamp.^60^

#### Thermodynamic or structural characteristics

In a comprehensive DNA duplex melting study by Heckel *et al.*, the *o*NB derivatives including NPP, NPE, NPOM, and NDBF were shown to generally lower the melting temperature (*T*_m_) of 15-mer DNA duplexes by 6–16 °C, which is also affected by sequence.^61^ Notably, the *T*_m_ of NPE groups in DNA duplexes was also sensitive to the configuration of the stereogenic center (indicated as * in Fig. 1): (*S*)-NPE group decreases *T*_m_ by 9.2 °C *versus* that of the unmodified sequence, a larger decrease relative to a 4.8 °C decrease by the (*R*)-NPE.^62^ NOE-based structural analyses revealed that both enantiomers retained Watson–Crick base pairing of the NPE-modified dC base and its partner, but the different NPE stereoisomers interacted with neighboring bases differently, resulting in the differential impact on its thermal stability.^62^ Min *et al.* showed *T*_m_ of NPOM-caged 24-mer duplex DNA is ∼7 °C lower than that of the unmodified DNA, while the *T*_m_ of NPOM-DNA after photocleavage was the same as that of the unmodified DNA.^58^ Molecular dynamics simulations of NPOM-dT containing DNA indicates that NPOM may occupy in the major groove of the DNA as the nucleobase takes up a *syn* conformation.^58^ Introduction of three NPOM groups over 14-bp duplexed region within a DNA hairpin, the melting temperature decreased by ∼30 °C.^9^ The impact of NPOM modification in U or G in RNA duplex (21-bp) also depended on the position and number of modifications.^47^ Heckel *et al.* also reported that NDBF on N^4^-dC and N^6^-dA in 15-bp DNA duplexes lowered the *T*_m_ by 16 °C and 12 °C compared with the unmodified duplexes, which were larger decreases than those caused by NPE modifications in the equivalent positions (Δ*T*_m_ = −8 °C and −6.2 °C).^54^

#### Biological applications

*o*NB family of modifications are the most versatile, and each was used to photo-regulate nucleic acid functions in various ways.

##### NPP and NPE modifications

NPP-dT and NPE-dT were used to block the binding of the MutS mismatch repair protein to a DNA bulge, which then could be removed by photoirradiation and enable the binding.^63^

NPP, NPE, and *o*NB linkers were also widely used as photocleavable linkers in the backbone of oligonucleotides to control gene expression and editing. For instance, NPE has been utilized in modulating siRNA activity.^64^ In a recent work, single-stranded RNA circularized *via* an *o*NB linker in the phosphodiester backbone was used as siRNA precursor, which could efficiently be activated to linear RNAs by 365 nm irradiation *in vitro*.^36^ Various types of *o*NB-based linkers were also used: *e.g.*, as internal photocleavable linkers within single guide RNA (sgRNA) to inactivate Cas9 nuclease and attenuate genome editing by CRISPR-Cas9 within cells^37,39^ and as a way to control RNA-cleaving DNAzyme's activity.^34^

##### NPOM

The ability of NPOM to disrupt DNA and RNA hybridization has been used in various applications such as DNA nano-tweezer,^60^ DNA triplex nanostructures,^65^ DNA computation,^66^ as well as controlling antisense DNA agent activity,^52^ DNAzyme activity,^50,67^ restriction endonuclease,^49^ and polymerase chain reaction.^68,69^ NPOM was also used as a part of gene promoters, triplex-forming oligonucleotides, microRNA, siRNA^47^ as a tool to regulate gene transcription and translation. In more recent works, NPOM was applied to the CRISPR-Cas9 gene editing system. In one study, NPOM-caged guide RNAs (gRNAs) conferred complete suppression of gRNA:dsDNA-target hybridization, which could subsequently be restored with light irradiation.^51^ In another study, NPOM-modified gRNA hybridized with DNA and allowed Cas9 to bind DNA, but the gene cleavage was suppressed until light-induced activation.^70^ This approach, referred to as very fast CRISPR (vfCRISPR), also created double-strand breaks (DSBs) at a submicrometer scale within seconds, which could be used to track the recruitment of DSB repair proteins to the damaged sites.^70^ Min *et al.* also showed that NPOM-modified dT could be specifically bound by the Rad4/XPC DNA nucleotide excision repair protein and that such binding was abolished upon light-induced photocleavage of NPOM.^58^ Other biological applications of NPOM include modulating mRNA splicing (splice switching) in cells and zebrafish^40^ and controlling TLR9 in immune responses.^18^

##### DMNPE & DMNEC

DMNPE was used to control the activity of siRNA as a part of the internal backbone or its termini.^56^ For instance, the regioselective incorporation of DMNPE groups in the four phosphate termini of an siRNA duplex effectively limited the RNAi activity, which could be restored upon irradiation.^56^ On the other hand, the DMNEC modifications were used to post-synthetically acylate 2′-hydroxyls of RNA ribose. Hammerhead ribozyme activity could be photo-regulated using this method in which multiple DMNEC groups were incorporated along the RNA molecule.^26^ Later, Zhou *et al.* used DMNEC on gRNA to suppress CRISPR-Cas gene editing, which could be reversed by 365 nm light.^57^

### 2. *p*-Hydroxyphenacyl

**Fig. 3:**
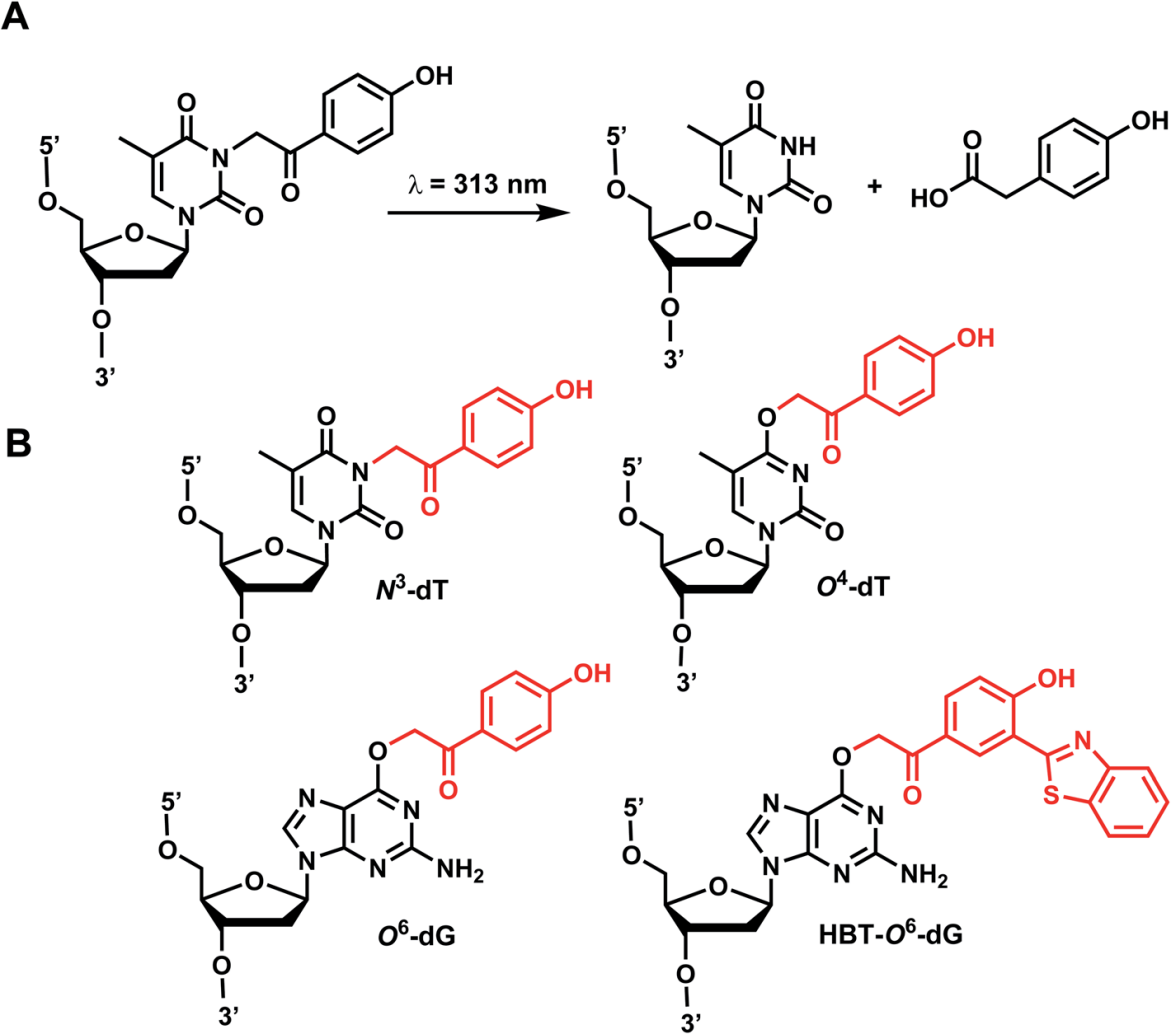
*p*HP modifications on oligonucleotides nucleobase. (A) Upon light irradiation, the *p*HP group undergoes skeletal rearrangements and is removed from the nucleobases of DNA and RNA, restoring the native structure. (B) *p*HP and *p*HP-derived modification on DNA nucleobases on N^3^-dT, O^4^-dT, O^6^-dG.

*p*-Hydroxyphenacyl (*p*HP) modification has been first introduced by the Reese group.^71^ Currently, *p*HP modification and their photolysis reactions have been reported for base modifications on N^3^-dT,^59^ O^4^-dT,^72^ and O^6^-dG (Fig. 3).^73^ Addition of a benzothiazole to *p*HP as 2-(2′-hydroxyphenyl)benzothiazole (HBT) has also been introduced as fast decaging moiety on O^6^-dG (Fig. 3B).^74^

#### Reaction characteristics

*p*HP photosolvolysis typically occurs far more rapidly following excitation compared with the more commonly used *o*NB derivatives (Section 1), which proceeds through an intermediate that can exist for seconds to a minute. The deprotection rate of *p*HP correlates inversely with the p*K*_a_ of the conjugate acid of the leaving group. The absorption spectrum also changes drastically as the reaction progresses from a conjugated phenyl ketone to a nonconjugated phenol, 4-hydroxyphenyl acetate.^11^

The photocleavage reaction of *p*HP on N^3^-dT was slow (1 h using 313 nm),^59^ but the incorporation of *p*HP in O^4^-dT shortened the photodecaging, and complete photodecaging was achieved in 0.3 min.^72^*p*HP at the O^6^-dG position was decaged with a time constant *t*_1/2_ of 17 s upon irradiation with 295 nm UV light.^73^ Later, Singh *et al.*^75^ improved the photodecaging reaction by synthesizing HBT. HBT has strong fluorescence and can use longer wavelength light (400 nm) for decaging. HBT-modification on O^6^-dG cleaved using blue light (405 nm) in a short pulse (≤30 ms) was used for rapidly initiating the folding and activation of twister ribozyme in a single molecule study.^74^

#### Thermodynamic or structural characteristics

*p*HP modifications are reported to be thermodynamically destabilizing for DNA duplexes. *p*HP-caged N^3^-dT or O^4^-dT can destabilize 15-bp duplexes DNA by ∼9 °C.^59,72^*p*HP-caged O^6^-dG is also proposed to prevent RNA annealing due to the steric hindrance and changes in the base-pair hydrogen-bonding patterns.^73^

#### Biological applications

*p*HP modifications were shown to temporarily block the antisense pairing between non-coding RNAs catalyzed by the RNA chaperone Hfq^62^ and to regulate the function of the twister ribozyme.^73,74^ The fast uncaging of *p*HP and its derivatives could be promising for various time-resolved studies that require the photoremoval reaction to occur faster than the molecular process under investigation.^73^

### 3. TEEP-OH (thioether-enol phosphate, phenol substituted)

**Fig. 4:**
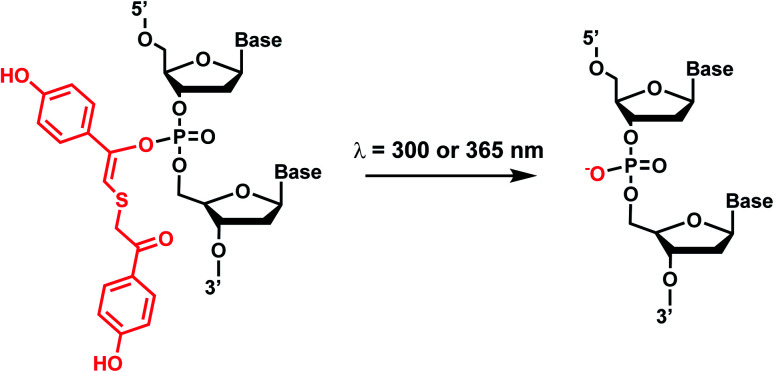
Light-induced photocleavage reaction of the TEEP-OH group from a phosphate backbone. Schematic of the photocleavage reaction. Upon light irradiation (*λ* = 300 or 365 nm), the TEEP-OH group is cleaved from the phosphate group in the DNA, restoring the DNA backbone.

Photolabile TEEP-OH (thioether-enol phosphate, phenol substituted) was inspired by the *p*-hydroxyphenacyl bromide group (*p*HP, Section 2)^76^ and can be incorporated *via* post-synthetic modification on the phosphodiester backbone of phosphorothioate DNA.^28^ Upon light irradiation, TEEP–OH is photocleaved and the phosphate backbone reverts to its native form (Fig. 4). This modification was used for photoregulation of an RNA-cleaving DNAzyme, a G-quadruplex peroxidase-mimicking DNAzyme, and a thrombin-binding aptamer.^27,28^

#### Reaction characteristics

The photodecaging of the RNA-cleaving DNAzyme was carried out using 365 nm light (12 W hand-held UV lamp) for 15 minutes.^28^ In a different application with G-quadruplex, the photoreaction was carried out by 300 nm light (12 W hand-held UV lamp) with the sample-to-lamp distance of 5 cm for 20 minutes.^27^

#### Thermodynamic or structural characteristics

Not reported.

#### Biological applications

TEEP-OH modification of RNA-cleaving DNAzymes in their active sites significantly inhibited the DNAzyme's activity. Upon light irradiation at 365 nm, the activities were restored to those of the native enzyme. The photodecaging and restoration of DNAzyme activity could also be accomplished when the DNAzyme and its substrates were transfected into HeLa cells.^28^ TEEP-OH modification of a G-quadruplex DNAzyme also inhibited the DNAyzme's peroxidase activity, which could be restored by UV photocleavage.^27^ TEEP-OH photocaging also could inhibit the activity of thrombin-binding G-quadruplex aptamer, which could be restored upon decaging by UV light.^27^

### 4. Aryl sulfide

Originally reported by the Greenberg group as a way to study nucleobase radical formation, electron-rich aryl sulfide (ArS, dimethoxythiophenyl) undergoes carbon–sulfur bond homolysis upon irradiation.^77^ ArS-modified nucleobase (*e.g.*, on C^5^-methyluridine or C^6^-hydrothymidine) disrupts nucleic acid structure by perturbing base stacking.^77,78^ ArS on 5-methyluridine prevents RNA hairpin formation in short RNA as well as the folding of the preQ1 class I riboswitch.^78,79^

#### Reaction characteristics

**Fig. 5:**
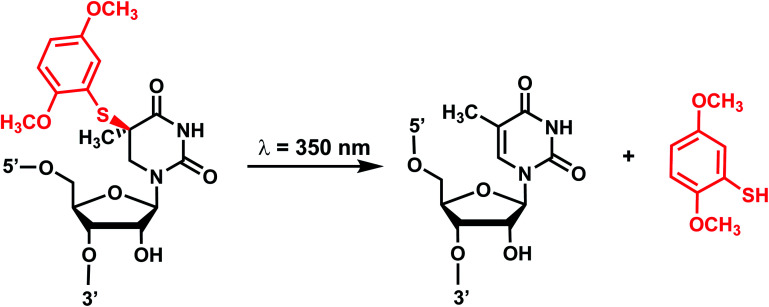
Light-induced photocleavage reaction of the ArS group from nucleobase. Schematic of the photocleavage reaction. Upon light irradiation (*λ* = 350 nm), the ArS group is cleaved, restoring 5-methyluridine in the RNA.

Photolysis produces a native nucleotide *via* a radical pair that undergoes disproportionation within a solvent cage upon irradiation with light at 350 nm (Fig. 5).^78^ The reaction was complete within minutes and the rate is estimated to be very fast, in the order of microseconds, based on thiol competition experiments.^78^

#### Thermodynamic or structural characteristics

ArS is shown to disturb the base-pairing and thus secondary structure of the A-form RNA hairpin, as monitored by CD spectrometry.^78^ ArS modification also thermodynamically destabilizes DNA and RNA duplexes.^77,78^ For instance, ArS-modified hydrothymidine in a 12-bp DNA duplex lowered the *T*_m_ by 10 °C.^77^

#### Biological applications

In studies by Greenberg *et al.*, the incorporation of ArS inhibited the folding of a preQ1 class I riboswitch that binds to the preQ1 ligand to form RNA pseudoknot.^78^ Such inhibition of RNA folding could be abolished upon the photocleavage of ArS.^78^

### 5. Nitroindole group

**Fig. 6:**
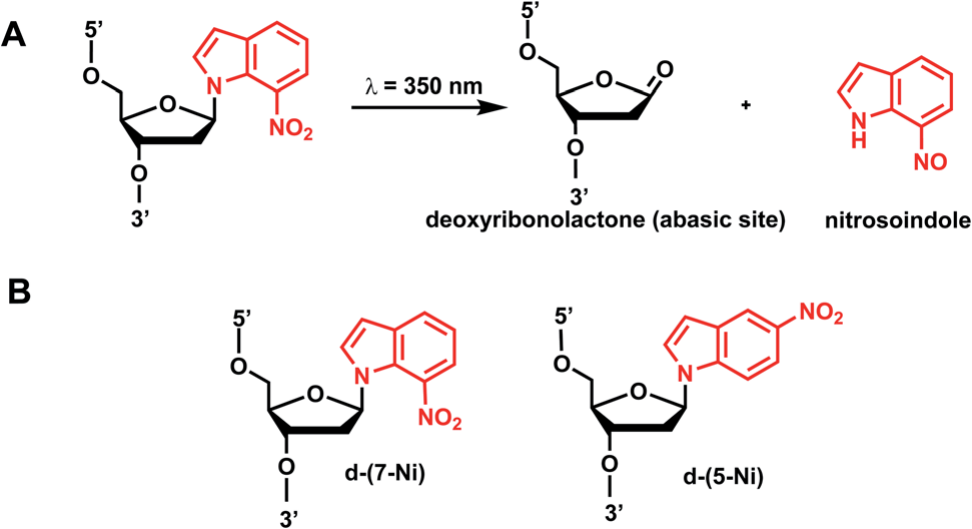
Nitroindole modification on oligonucleotides. (A) Upon light irradiation, the nitroindole group is cleaved from the ribose in the DNA and generating abasic lactone and nitrosoindole. (B) Nitroindole-derived modification on DNA deoxyribose 7-nitroindole (d-(7-Ni)), 5-nitroindole (d-(5-Ni)).

Photocleavable nitroindole nucleoside was introduced by Lhomme and colleagues.^80,81^ Irradiation (*λ* = 350 nm) of oligonucleotides containing 7-nitroindole or 5-nitroindole triggers a radical process: the excited nitro group induces an intramolecular H1′ abstraction leading to the release of a nitrosoindole group while forming a highly labile deoxyribonolactone, an abasic site (Fig. 6).^82^ Subsequent mild basic or thermal treatment leads to cleavage of the DNA backbone *via* β- and δ-elimination at the abasic site.^82^

#### Reaction characteristics

The photocleavage reactions of nitroindole-modified DNA nucleoside accompany changes in the absorption spectra. Two isobestic points at 310 nm and 365 nm were observed in the UV spectra of the irradiated solution of the free nucleoside, which is monitored and characterized by nitrosoindole (*λ*_max_ = 406 nm) and deoxyribonolactone (*λ*_max_ = 241 nm) formation in different time intervals. Photolysis of 7-nitroindole-containing oligonucleotides with 350 nm UV-A light was completed in a few minutes (*t*_1/2_ = 1.0 min).^83^

#### Thermodynamic or structural characteristics

7-Ntroindole and 5-nitroindole DNA nucleosides have both been shown to lower the *T*_m_ of DNA duplexes. 7-Nitroindole was slightly more destabilizing than 5-nitroindole:^82^ 13–15 °C lower *T*_m_ for 7-nitroindole than that for unmodified DNA *versus* 10–11 °C lower *T*_m_ for 5-nitroindole in the same 11-bp DNA duplex context.^82^

#### Biological applications

Light-induced photocleavage of nitroindole was used to induce controlled release of DNA-binding proteins such as NF-κB as a part of the catch-and-release DNA decoys where 7-nitroindole could be used in place of regular dG's in the NF-κB binding sequence.^83^

### 6. Benzophenone and acetophenone

**Fig. 7:**
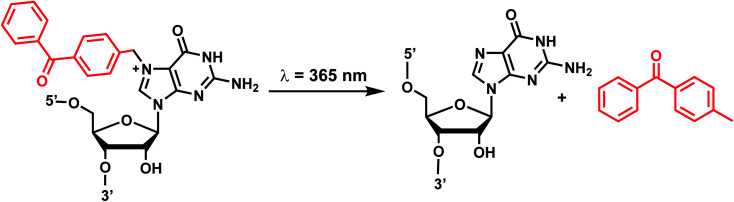
Schematic of benzophenone cleavage from N^7^-G in an RNA nucleoside upon light irradiation at *λ* = 365 nm.

**Fig. 8:**
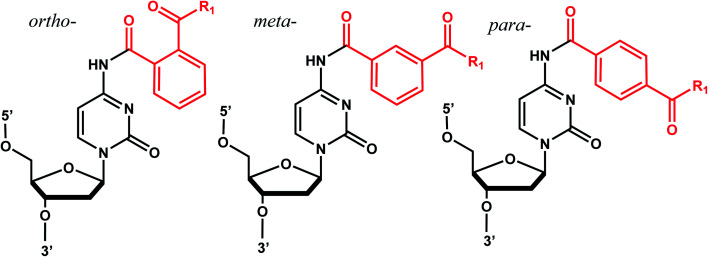
Acetophenone (R_1_= methyl) and benzophenone (R_1_= phenyl) groups as crosslinkers. Acetophenone and benzophenone modified N^4^-dC nucleosides. These modifications can be installed in *ortho*, *meta*, and *para* positions of the benzene ring. The C–C crosslinking occurs between the C of the carbonyl group in phenones and a Cα of the peptide bond.

Benzophenones contain a carbonyl carbon that undergoes intersystem crossing in high yields, making it a robust triplet photosensitizer for use in organic and biological chemistry.^84^ While a wide range of applications have utilized its light-induced C–C photocrosslinking properties,^85^ the Rentmeister group reported that benzophenone modification at N^7^-G in the context of RNA oligonucleotides can undergo a photocleavage reaction (Fig. 7).^86^ Upon irradiation with 365 nm light, benzophenone is cleaved from nucleobase through hydrogen abstraction mechanism. The photocaged guanosines used as a 5′-cap blocked the RNA's interactions with the translation initiation factor eIF4E and the RNA decapping enzyme DcpS.^86^ Benzophenones and acetophenones also can form UV-induced (C–C) cross-links with protein amino acids.^84^ For example, terminal deoxynucleotidyl transferase enzyme could be crosslinked to 3′-tails of DNA containing benzophenones and acetophenones on N^4^-dC's using 365 nm light (Fig. 8)^87^

#### Reaction characteristics

UV irradiation (365 nm) of benzophenones generates C–O biradical through n–π* transition, which can lead either to photocrosslinking or its reversal, photocleavage.^84,85^ The photocleavage reaction was rendered complete after 10 min of irradiation at 365 nm.^86^

#### Thermodynamic or structural characteristics

None reported.

#### Biological applications

Benzophenone modification of an N^7^-G blocked the interaction between the 5′ cap in the mRNA and the translation initiation factor eIF4E and the mRNA-decapping enzyme DcpS. Photocleavage followed by remethylation of the N^7^-G in 5′ cap (GpppA to *m*^7^GpppA) restored the binding with these proteins.^86^ On the other hand, benzophenone- and acetophenone-modifications on N^4^-dC could generate protein-DNA crosslinks with the bound terminal deoxynucleotidyl transferases using 365 nm light.^87^

### 7. Coumarin

**Fig. 9:**
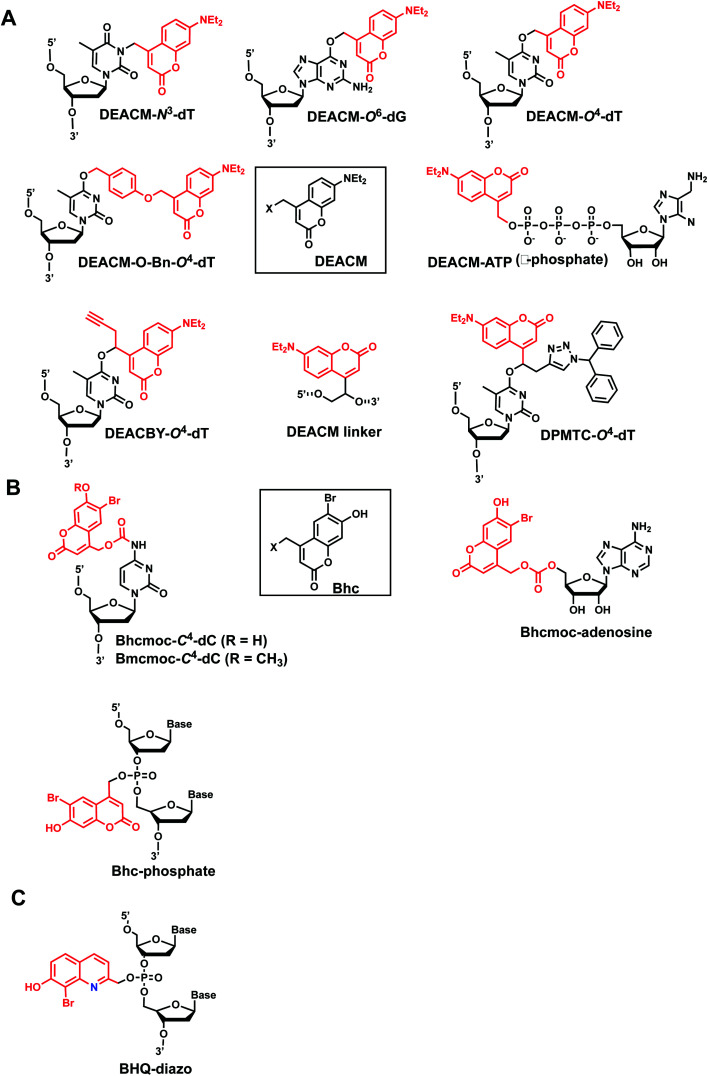
Representative coumarin-derived modification on oligonucleotides. (A) DEACM and (B) Bhc modifications in various positions of oligonucleotides. (C) Quinoline-based BHQ-diazo modification on the phosphodiester backbone.

Coumarin-based groups are widely used photo-removable groups because of their large molar absorption coefficients at longer wavelengths, high release rates, and fluorescent properties. They are also capable of photo-crosslinking *via* [2 + 2] cycloaddition. One of the representative coumarin derivatives, (7-diethylaminocoumarin-4-yl)methyl (DEACM) was first introduced by Hagen *et al.*^88^ (Fig. 9). DEACM could be introduced in the backbone of the DNA or on N^3^-dT,^72^ O^6^-dG,^89^ and O^4^-dT (without and with a linker as in DEACM-O-Bn-dT)^90,91^ as well as on the γ-phosphate group of ATP (Fig. 9A).^92^ 6-bromo-7-hydroxycoumarin-4-ylmethyl (Bhc) was first introduced by Tsien *et al.*^93^Bhc has been employed in modifying C^4^-dC (as Bhcmoc or Bmcmoc),^94^ 5′-position of the ribose in adenosine,^95^ and the phosphate backbone (Fig. 9B).^96,97^ The mechanism for photo-removal of these coumarin derivatives is through solvent-assisted photo-heterolysis (S_N_1 mechanism). Coumarin-modified oligonucleotides have been applied in various ways, for instance, in generating DNA strand breaks (*e.g.*, by using DEACM linker in Fig. 9A) and in regulating DNA polymerization, translation and transcription. Inspired by Bhc, a series of photo-removable groups based on quinoline was reported by Guo *et al.* Among them, 8-bromo-2-diazomethyl-7-hydroxyquinolinyl (BHQ-diazo) showed the highest caging and restoration efficiency for the anti-thrombin aptamer HD1 (Fig. 9C).^98^

#### Reaction characteristics

**Fig. 10:**
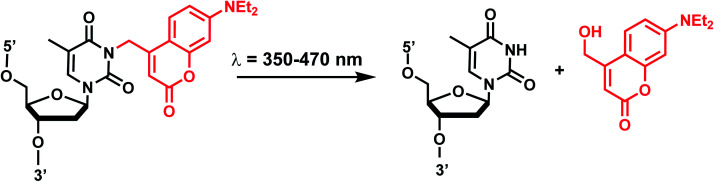
Coumarin photocleavage reaction. Upon irradiation with 350–470 nm light, the DEACM group is cleaved from the nucleobase dT, which restores the native structure.

The photocleavage reactions of coumarin derivatives can be accomplished by a broad range of light wavelengths (350–470 nm) (Fig. 10).^6,94,95,99^ Reported reaction times range from seconds to minutes.^59,72,89,94,95^ The presence of the extended spacer in DEACM-O-Bn-dT leads to fast decay and no byproduct.^90^DEACM-containing oligonucleotides exhibit a very intensive red-shifted absorption band (*λ*_max_ = 398 nm) compared with *o*NB (*λ*_max_ = 365 nm), from the π–π* transitions of the coumarin chromophore.^89^ (*S*)-diphenylmethyltriazole-coumarin (DPMTC) O^4^-dT derived from post-synthetic Cu-catalyzed azide–alkyne on DNA (*i.e.*, click chemistry) could be uncaged with 405 nm light within minutes.^95,103^ For the quinoline-derivative BHQ, photolysis occurs through solvent-assisted photoheterolysis (S_N_1) reaction mechanism as with the coumarin family.^100^ Light irradiation at 365 ± 5 nm with an approximate dose of 100 mJ cm^−2^, caged anti-thrombin aptamer HD1 was released more than 90% within 30 s.^98^

**Fig. 11:**
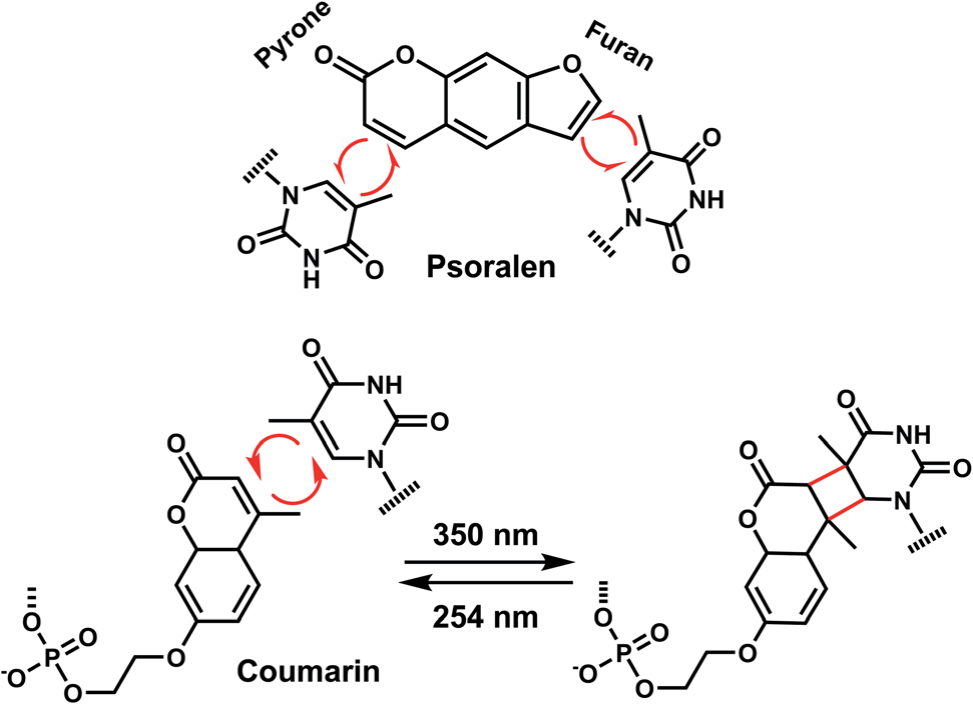
Light-induced reversible [2 + 2] cycloaddition of the Coumarin group. Schematic of the ICL formation upon light irradiation (*λ* = 350 nm), and reversible reaction (*λ* = 254 nm).

In addition to the photocleavage reaction, coumarin molecules can also undergo reversible photocrosslinking *via* a photo-induced [2 + 2] cycloaddition reaction similarly as psoralen (Fig. 11).^101,102^ The photocyclization reaction between coumarin and thymidine leads to fast and quantitative DNA interstrand crosslink (ICL) formation (>98%).^101^ The DNA crosslinks were generated by 350 nm irradiation whereas the reverse reaction, cyclo-reversion of the photo-adducts were achieved by 254 nm light (Fig. 11).^101^ ICL formation between a coumarin moiety containing a flexible two-carbon or longer chain and thymidine on the opposite strand completely quenches the fluorescence of coumarin, which allows for the monitoring of DNA crosslinking process over time *via* fluorescence spectroscopy.^101^ DNA crosslinking by coumarins shows a kinetic preference when flanked by an A:T base pair as opposed to a G:C pair.^101^

#### Thermodynamic or structural characteristics

In a comprehensive DNA duplex melting study by Heckel *et al.*, the coumarin derivatives (without crosslinking) were shown to generally destabilize the DNA duplex.^103^DEACM-N^3^-dT in a 15-bp DNA duplex showed a decrease in *T*_m_ of 13.5 °C (Δ*T*_m_ = −13.5 °C) and (*S*)-DPMTC-O^4^-dT showed Δ*T*_m_ of −15.8 °C, which are among the largest destabilization values reported for the coumarin family of modifications.^72,103^DEACM-O^6^-dG also decreased the melting point by 11.3 °C in a 15-bp DNA duplex.^89^

#### Biological applications

DEACM-incorporated photocleavable linker was used to catch and release NF-κB, a DNA-binding transcription factor, whereby photocleavage (365 nm) and subsequent DNA strand break abrogated the binding.^99^DEACM-ATP used as photocaged ATP: its uncaging *via* remote light (400 nm) was used for transient DNA polymerization.^92^DEACBY alkyne was used to inhibit duplex formation between a circular DNA and its target, which could be restored upon light irradiation.^91^Bhc-caged mRNA has been used to control the translation activity of mRNA *in vitro* and *in vivo*. Illumination with 350–365 nm ultraviolet light removed Bhc from caged mRNA, resulting in a recovery of translational activity.^96^ Also, BHQ-diazo as a modification on the phosphate backbone group of a 15-bp anti-thrombin aptamer HD1 inhibited the thrombin binding, which could be restored upon light irradiation.^98^ Applications of coumarin-based DNA crosslinking in a biological context remains to be seen.

### 8. Carbazole

**Fig. 12:**
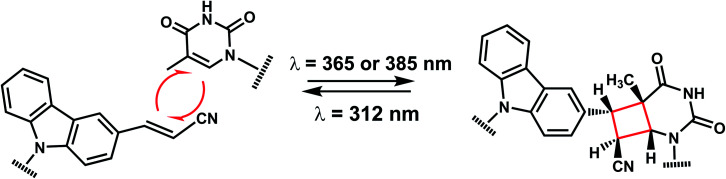
Reversible [2 + 2] reaction in ^CNV^K. 3-cyanovinylcarbazole nucleoside (^CNV^K) can undergo rapid photo-crosslinking to the complementary strand at one wavelength. Rapid reversal of the crosslink is also possible at a second wavelength.

^CNV^K nucleoside was further developed to improve photoreactivity. 3-Cyanovinylcarbazole-modified d-threoninol (^CNV^D) which has a flexible structure showed enhanced photoreactivity for the pyrimidine base at the −1 position in the complementary strand (Fig. 13): the photoreactivity of ^CNV^D was 1.8- (for crosslinking with dT), 8- (with dC), and 2.8-fold (with U in RNA) greater than that of ^CNV^K.^107^

The photo-crosslinking has been widely used to stabilize complexes with DNA by a covalent-bond formation.^104,105^ For instance, photo-crosslinkers such as psoralen (a member of furocoumarin family, related to coumarin, Fig. 11) can produce DNA inter-strand crosslinks *via* [2 + 2] cycloaddition reaction when irradiated by UV-A (365 nm) either *via* their furan or pyrone photoreactive site. The crosslinks can be reversed upon irradiation 254 nm shorter wavelength. Although psoralen is widely used as a thymine-selective photo-crosslinker in biological studies, there are limitations such as requiring a TpA step in the sequence and causing photodamage to DNA by forming pyrimidine photodimers upon cycloreversion that uses UV-C (254 nm).^104,106^ To alleviate these issues, Fujimoto *et al.*, reported 3-cyanovinylcarbazole nucleoside (^CNV^K) as a reversible photo-crosslinker that can photo-crosslink to pyrimidine base located 5′ to the complementary base through [2 + 2] cycloaddition with 385 or 365 nm irradiation (Fig. 12).^106^ The resulting photo-adducts can be uncrosslinked by 312 nm irradiation without causing DNA damage.^106^^CNV^K shows higher reactivity compared with psoralens, showing 97% yield with 1 s of longer wavelength UV-A light (366 nm).

**Fig. 13:**
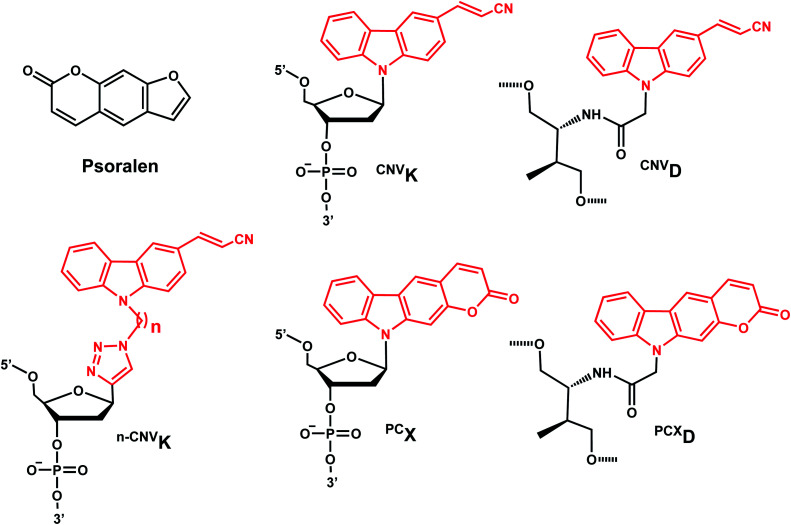
Carbazole derivatives. Both ^CNV^K and ^PC^X carbazole derivatives are inspired from psoralen. Either the vinyl in ^CNV^K, ^CNV^D, ^*n*-CNV^K or the pyrone in ^PC^X, and ^PCX^D undergo reversible [2 + 2] photoreaction.

^PC^X, pyranocarbazole nucleoside was developed to use visible light instead of UV-A, therefore less toxic and damaging (Fig. 13).^14^ Recently d-threoninol version of the ^PC^X photo-crosslinker (^PCX^D) was reported, showing a higher photoreactivity than ^PC^X (Fig. 13).^8^ In addition, *^n^*^-CNV^K with variable linker lengths (*n* = 2–5) was developed to use with click chemistry.^108^ This probe is capable of photo-crosslinking with pyrimidine bases at locations other than the −1 position (Fig. 13).^108^

#### Reaction characteristics

^CNV^K, ^CNV^D, ^PC^X, and ^PCX^D can photo-crosslink to pyrimidine bases within a few seconds of photoirradiation. NMR, kinetic, and structural analysis indicated that the photo-crosslinking reaction with thymine proceeds with *trans* isomer of ^CNV^K, and one single photo-adduct. However, these photo-crosslinkers can only crosslink to the counter base if it is adjacent to the 5′-side (−1) position to the crosslinker-containing base (5′-nXn-3′ and 5′-Ynn-3′ where X is the crosslinker-containing base and Y indicates the position of the crosslinked pyrimidine).^8,14,106–112^

Fujimoto *et al.* reported that the rate constant of the photo-crosslinking reaction of ^CNV^D is 0.106 s^−1^ which is comparable to ^CNV^K (0.059 s^−1^). Within the same sequence, psoralen showed a much slower photo-crosslinking rate (0.003 s^−1^) using the same wavelength (365 nm).^110^ The reaction rate constant of ^PCX^D with cytosine is 4.3-fold larger than that of ^PC^X.^8^

#### Thermodynamic or structural characteristics

Molecular modeling studies indicated that photochemical [2 + 2] cycloaddition is facilitated with the orientation of the vinyl group of ^CNV^K to be stacked onto the C5–C6 double bond of pyrimidine nucleobases located at the −1 position on the complementary strand (see above).^104,111^ Overall, crosslinking stabilizes the oligonucleotide duplexes.^14,108,111^ The impacts of the photoconvertible groups on duplex stabilities before crosslinking reaction were also investigated by the Fujimoto group. In general, a flexible threoninol linker (*e.g.*, ^CNV^D) destabilizes the duplex than having a regular phosphodiester linker with deoxyribose (*e.g.*, ^CNV^K): in a 9-bp DNA duplex, ^CNV^D was more destabilizing than ^CNV^K by 5 °C.^8^ Also, pyranocarbazole (*e.g.*, ^PC^X or ^PCX^D) is more destabilizing than 3-cyanovinylcarbazole (*e.g.*, ^CNV^K and ^CNV^D). ^PCX^D-containing duplex has the lowest melting compared to the others.^8^

#### Biological applications

Vinylcarbazole-based photocrosslinkers have been used for applications such as targeted site plasmid labeling,^113^ transient transgene silencing,^114^ and identifying targets of endogenous small RNAs.^115^^CNV^K and ^PC^X have been used for detecting locations of RNA,^7,116^ and methylcytosines in DNA in cells by fluorescence *in situ* hybridization (FISH). Incorporating multiple crosslinkers could help increase the sensitivity of FISH by 40-fold in the region where detection was difficult due to complex secondary structures using conventional FISH.^112^

Fujimoto group reported the use of ^CNV^K photocrosslinking in antisense DNA technology: photocrosslinkable antisense oligonucleotides containing ^CNV^K can regulate GFP expression in a sequence-specific manner only after 10 s photocrosslinking with 365 nm light in HeLa cells. In a recent study, they investigated and compared the photo-crosslinking rate and its inhibitory effect including ^CNV^D, ^CNV^K, and psoralen on gene expression.^110^ The inhibitory effect on gene expression was the highest with ^CNV^D (93%), while no inhibitory effects were observed with psoralen.

In another recent study, they regulated the DNAzyme activity by photoirradiation through the photochemically reversible formation of covalent bonds.^109^ While photo-crosslinking using 365 nm completely abolished the activity of the DNAzyme harboring ^CNV^K, uncrosslinking using 312 nm irradiation restored the activity.^109^ C^NVK^ also was shown to accelerate *in vitro* DNA strand displacement reactions, which may be employed for rapid-response DNA nano device technology using higher-order DNA structures.^117^

### 9. Vinyl derivatives

**Fig. 14:**
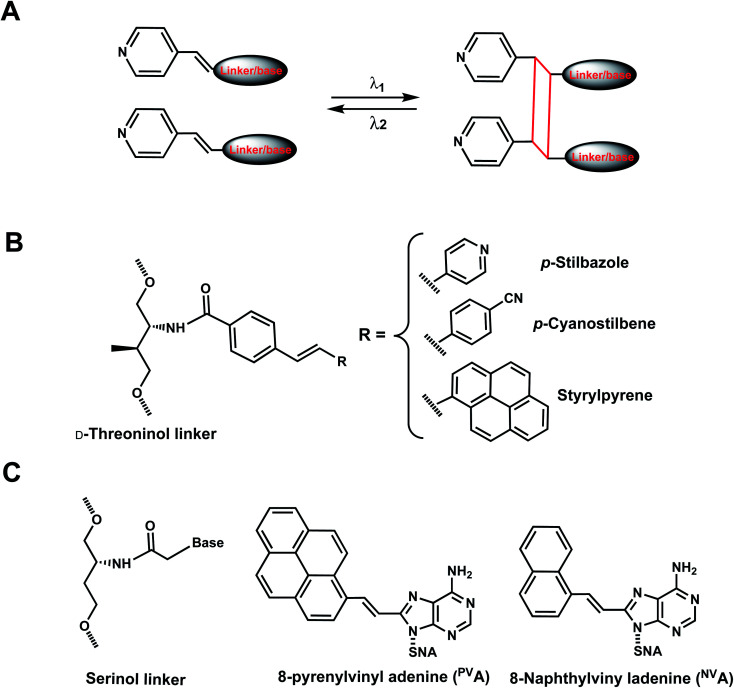
Vinyl derivatives for light-induced [2 + 2] cycloaddition. (A) Schematic of the [2 + 2] photo cycloaddition and its reverse reaction (cycloreversion) upon light irradiation in two different wavelengths. Usually, the wavelength for cycloaddition (crosslinking) is longer than that for the reverse reaction (un-crosslinking). (B) d-Threoninol linkers can be used with *p*-stilbazole, *p*-cyanostilbene, and styrylpyrene as a part of DNA backbone. (C) Serinol nucleic acid (SNA) linkers can also be used to introduce ^PV^A and ^NV^A to the oligonucleotides.

**Fig. 15:**
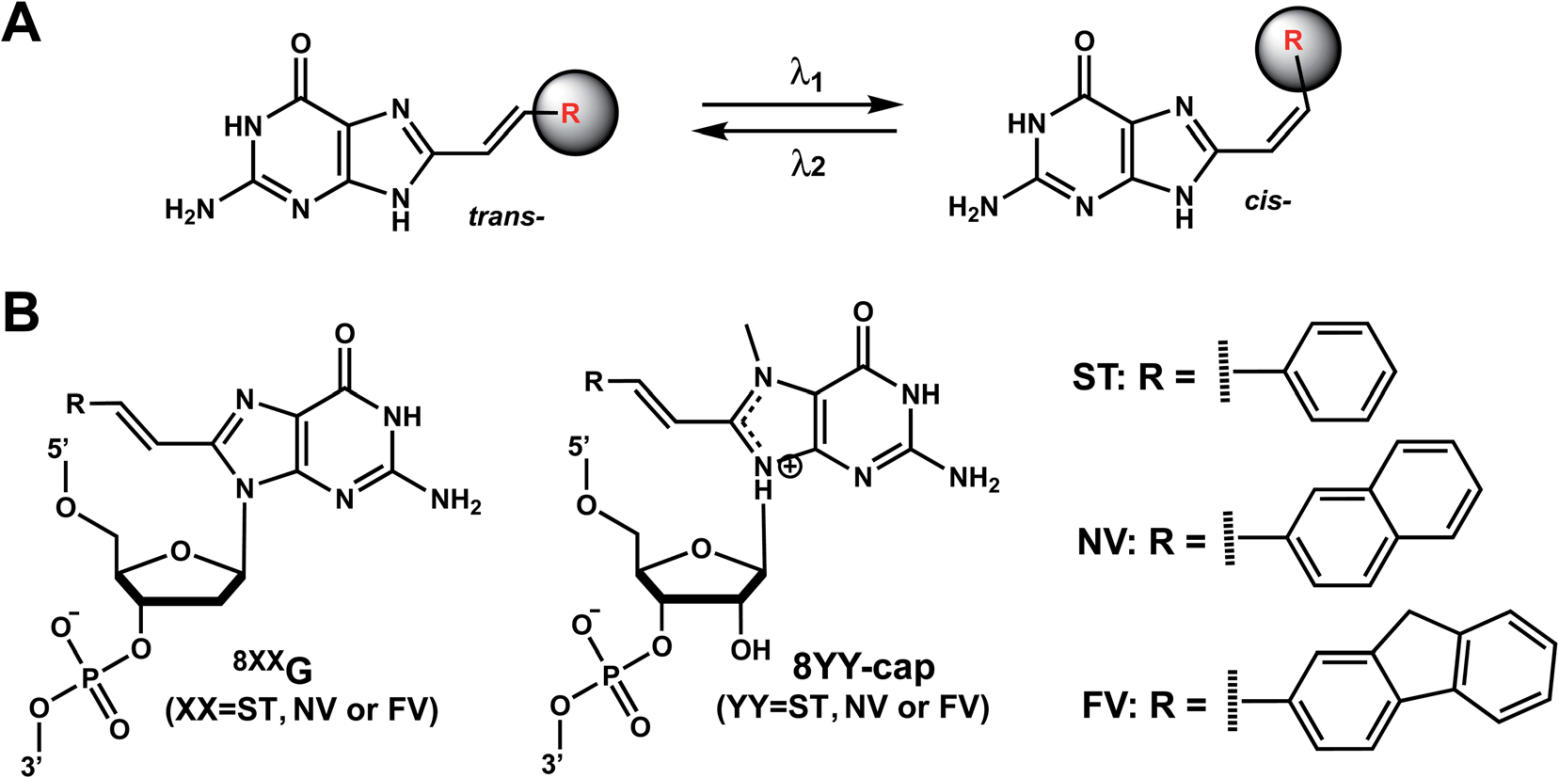
Vinyl group derivatives undergo reversible *trans*–*cis* photoisomerization. (A) *trans*-to-*cis* and *cis*-to-*trans* photoisomerization are catalyzed by two different wavelengths. (B) 8-styryl (8ST), 8-naphthylvinyl (8NV), and 8-fluorenylvinyl (8FV) modifications can be attached to C^8^-dG in DNA (“^8xx^G”) or to C^8^-methylG-cap in mRNA cap (“8YY-cap”) and can undergo *trans* to *cis* photoisomerization.

As with the vinyl groups in 3-cyanovinylcarbazole (CNV) modifications (Section 8), other DNA/RNA modifications containing vinyl group have been reported with photo-crosslinking properties (Fig. 14A). In addition, some vinyl-derivatives can also undergo *cis*- to *trans*-photoisomerization around the C–C double bond in the absence of a crosslinkable partner; this then alters the orientations of the attached moieties and subsequently the DNA/RNA structures (Fig. 15A).^118^ Here, we discuss the vinyl derivatives reported for these two reaction categories.

#### 9A. Vinyl derivatives for photocrosslinking *via* [2 + 2] cycloaddition

*p*-Stilbazole photo-dimerization was first illustrated by Asanuma *et al.*^119^*p*-Stilbazole positioned opposite of each other in DNA duplex, linked through d-threoninol linkers could be crosslinked with UV light, thus significantly stabilizing the duplex (Fig. 14B). NMR analyses indicated that two diastereomers are produced on photo-crosslinking due to rotation of vinyl group.^119^ Later, a stilbene derivative, *p*-cyanostilbene, was introduced at the termini of siRNA in both strands and photo-crosslinking resulted in “termini-free” siRNAs which could not be cleaved by dicer that requires 3′ overhang ends from the precursor siRNA (Fig. 14B).^120^ Similarly, styrylpyrene (Sp) pairs introduced in complementary positions in DNA duplexes could undergo a [2 + 2] cycloaddition photocrosslinking reaction by visible light irradiation (*λ* = 455 nm) whereas UV light (*λ* = 340 nm) could reverse the crosslinks (Fig. 14B).^121^

More recently, 8-pyrenylvinyl adenine (^PV^A) was employed as a way to control duplexation between serinol nucleic acid (SNA) and RNA (Fig. 14C). When incorporated in SNA in adjacent positions, ^PV^A could undergo intrastrand photodimerization by 455 nm light, which abolished the duplexation with a complementary RNA. However, the crosslinks could be reversed with cycloreversion catalyzed by 340 nm light.^122^ It is noteworthy that both the forward and reverse reactions could be carried out to completion at constant room temperature.^122^ 8-Naphthylvinyladenine (^NV^A) is also used in SNA for crosslinking/uncrosslinking reaction, similarly as ^PV^A, but uses a shorter wavelength of light than ^PV^A: intrastrand crosslink by irradiation with 340–405 nm light and reverse reaction by ≤300 nm light. In an SNA strand with adjacent ^NV^A and ^PV^A residues, irradiation with 405–465 nm led to intrastrand crosslink, which was reversed by irradiation with ≤340 nm light.^123^ In all these cases, the intrastrand photo-crosslinking destabilize SNA/RNA duplexes, resulting in duplex dissociation while its cycloreversion led to duplex formation.^123^ With these ^NV^A/^NV^A and ^NV^A/^PV^A photo-switches, the hybridization states of SNA/RNA duplexes could be independently controlled by using light of varying wavelengths.^123^

##### Reaction characteristics

Strylpyrene pairs (Spa and Spb) introduced as a part of d-threoninol linker in the opposite strands of DNA duplex could also undergo [2 + 2] photocycloaddition using visible light (*λ* ≈ 455 nm), but gave two diastereomers as a result of the rotation of the styrylpyrene residues.^121^ The reaction progress and stacking of the Sp dimers could be monitored using UV-Vis absorption spectroscopy. Upon visible light irradiation of S_P_a/S_P_b at *λ* = 455 nm, the absorption band at *λ* ≈ 390 nm decreased with irradiation time and almost disappeared after 60 min of irradiation while new bands concurrently appeared at *λ* = 338 and 354 nm. The progress of photocycloaddition was apparent also due to changes in color and fluorescence of the solutions from colored to colorless.^121^

^PV^A-containing oligonucleotide features an absorption band at around 400 nm (characteristic of vinylpyrene) and upon irradiation with 455 nm light immediately decreased and almost disappeared after 2 min using 203 mW cm^−2^ power.^122^ Simultaneously, new bands appeared at 270 and 354 nm, which correspond to absorption bands of alkylpyrene, a product of crosslinking. Upon irradiation of the crosslinked product with 340 nm light, the initial absorption bands were restored, indicating the recovery of ^PV^A monomers.^122^ The crosslinking and uncrosslinking reactions were rendered complete after 1 h (irradiation at 455 nm) and 15 min (340 nm), respectively.^122^

In the case of ^NV^A, irradiation with 405 nm light for 60 s led to the disappearance of the absorption band around 360 nm, and irradiation with 300 nm light of this photo-adduct led to the cycloreversion: 61% of the initial absorption band was recovered within 120 s.^123^ Four hybridization states of two SNA/RNA duplexes containing either the ^NV^A/^NV^A pair or ^NV^A/^PV^A could be orthogonally controlled using different wavelengths of light.^123^

##### Thermodynamic or structural characteristics

Photocrosslinking between Sp groups is thermodynamically stabilizing for DNA duplexes, as expected. Melting measurements revealed that both diastereomer products after crosslinking had melting temperatures significantly higher (22–25 °C) than that of the uncrosslinked dimer, S_P_a/S_P_b. Melting measurements also indicated that the crosslink had been reversed upon cycloreversion.^121^

^PV^A slightly destabilized the duplexes when compared with the unmodified SNA/RNA duplex.^122^ On the other hand, ^NV^A in SNA/RNA duplex slightly increased *T*_m_ compared with the control SNA/RNA^123^while ^NV^A intrastrand photo-crosslink caused severe destabilization of a SNA/RNA duplex containing ^NV^A or ^PV^A: this resulted in the melting of the duplex to single strands. The reverse reaction, cycloreversion, led to the restoration of the duplexes.^123^

##### Biological applications

^NV^A and ^PV^A were studied as a part of SNA/RNA as mentioned above as reversible crosslinkers that stabilize the SNA/RNA duplexes.^121,123,124^

#### 9B. Vinyl derivatives undergoing *cis*–*trans* photoisomerization

Several vinyl-containing modifications have been developed to modulate DNA and RNA oligonucleotides by reversible *cis*–*trans* photoisomerization (Fig. 15A). Maeda *et al.* synthesized three C^8^-substituted 2′-deoxyguanosine (dG) with vinyl-containing modifications to modulate DNA hybridization by reversible *cis*–*trans* photoisomerization: 8-styryl (8ST), 8-naphthylvinyl (8NV), and 8-fluorenylvinyl (8FV) (Fig. 15B).^125^ Rapid and efficient light-induced *trans*-to-*cis* isomerization led to changes (1.4–8 °C) in the thermal stability of the duplexes even at room temperature.^125^ These nucleosides in the *trans* forms have little influence on the B-form structure when duplexed, and their intrinsic fluorescence can be used to monitor the isomeric states since the fluorescence intensity dramatically changes upon *cis*–*trans* isomerization. For instance, the fluorescence emission maximum at 450 nm for *trans*-^8ST^G is 6 times higher than *cis*-^8ST^G.^125^^8FV^G was used for reversible photo-regulation of G-quadruplex aptamers to bind with thrombin through *cis*–*trans* photoisomerization.^126^ Later, the same modifications were attached to 5′-cap methylguanosine (methylG) of mRNA: 8ST-cap, 8NV-cap, and 8FV-cap were developed (Fig. 15B).^118^ 8NV-cap and 8ST-cap were used to reversibly regulate gene expressions.^118,127^

##### Reaction & thermodynamic characteristics

In 12-bp duplexes containing ^8ST^G, ^8NV^G, or ^8FV^G, the *trans* forms of the dG modifications were photoisomerized to the corresponding *cis* forms when irradiated for 5 min with 370, 410, and 420 nm light with 86%, 63%, and 77% conversion efficiencies. In addition, subsequent irradiation for 2 min at 254, 290, and 310 nm yielded the *trans* forms with 94%, 87%, and 77% conversion efficiencies, respectively.^125^

Thermal stability study of 12-bp duplexes containing ^8ST^G, ^8NV^G, or ^8FV^G showed both the *cis* and *trans* isomers were thermally stable. ^8ST^G-containing duplex showed the *T*_m_ of the *trans* form was 7.9 °C higher than that of the *cis* form. This is probably due to a difference in the steric hindrance of the benzene ring with its neighboring nucleobase and backbone. In contrast, the *T*_m_ of the *trans* forms of ^8NV^G- and ^8FV^G-containing duplexes were only 1.6 and 1.4 °C lower than the *cis* forms, respectively.^125^ This may indicate that the bulky substituents, naphthalene and fluorene in ^8NV^G and ^8FV^G, may cause serious steric hindrance with the backbone, even in the *trans* form.^125^

##### Biological applications

The *trans* to *cis* isomerization of vinyl derivatives can regulate oligonucleotide duplex hybridizations^125^ and has been applied in various biological applications.^101,127^ For example, The mRNA containing the 8NV-cap at the 5′-end could be switched between a translating (ON) state when in *cis* form and a non-translating (OFF) state when in the *trans* form in a reversible fashion by alternately irradiating with 410 nm or 310 nm light.^118^ In addition, 8ST-cap can reversibly regulate translation by controlling the interaction with eukaryotic translation initiation factor eIF4E through its *cis*–*trans* photoisomerization in living mammalian cells as shown in PC12 neuronal cell line through its neurite expansion and contraction.^127^ Furthermore, trans (*E*)-to-cis (*Z*) photoisomerization of the ^8ST^G was utilized by Zhou *et al.* to reversibly switch between a B-form and Z-form DNA by alternately illuminating with monochromatic 254 nm and 365 nm light.^128^

### 10. Azobenzene

**Fig. 16:**
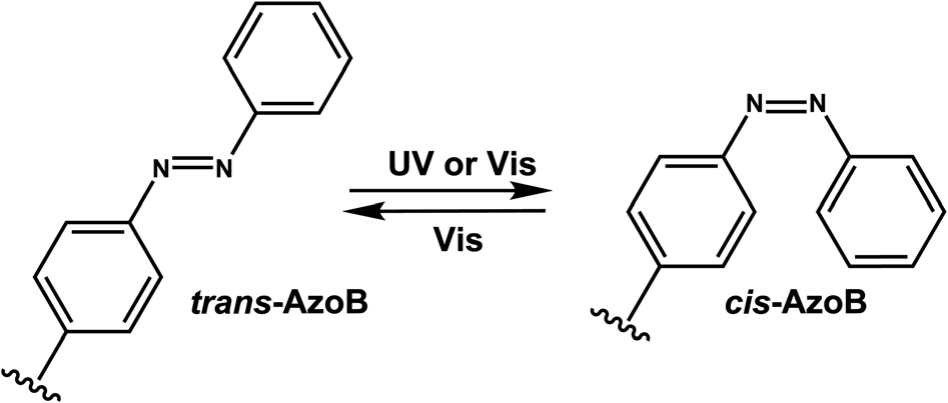
Schematic of reversible azobenzene *trans*–*cis* photoisomerization with different wavelengths. This conversion occurs through reversible rotation of planar *trans* N=N to non-planar *cis* isomer. *λ*_1_ is often in the UV-A range, and *λ*_2_ in the visible range.

Azobenzenes (AzoB) are the most widely used *reversible* photoswitches in oligonucleotides due to their high quantum yields, fast switching, low rate of photobleaching, easy synthesis, high fatigue resistance (high repeatability of photoswitching), and good thermal stability.^12^ Irradiation with UV light converts planar *trans*-N=N bond with zero dipole to non-planar *cis* isomer (dipole moment of ∼3 D), which can be accelerated by heat (Fig. 16).^129^ The reverse *cis* to *trans* isomerization can be achieved by visible light.^129^ AzoB was first introduced in nucleic acids as a part of a flexible backbone linker based on propionic acid by Asanuma *et al.*^130^ and was used to regulate duplex^131^ and triplex^5,132^ DNA formation.

**Fig. 17:**
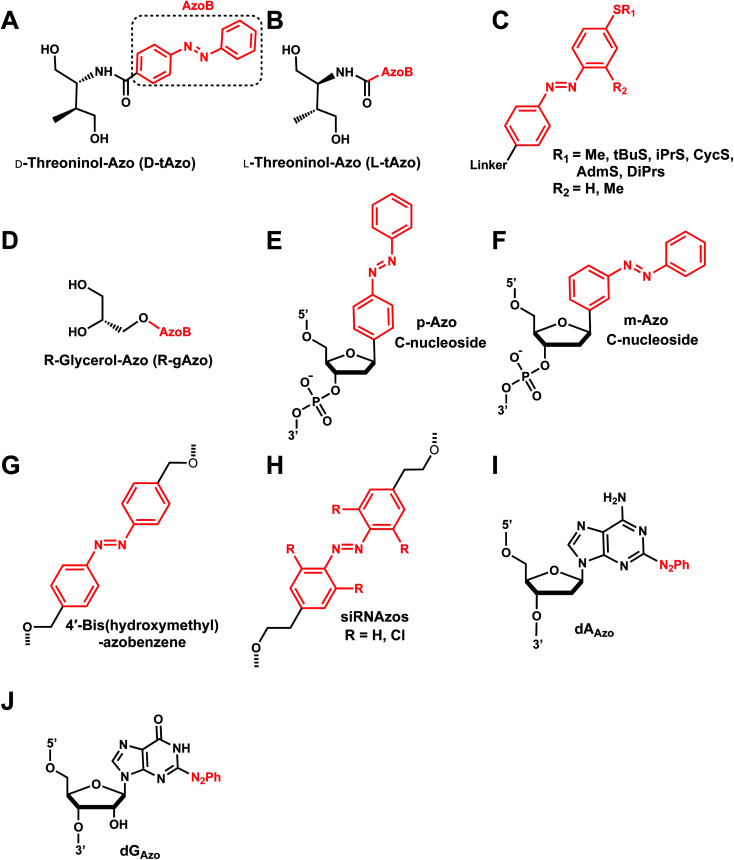
Azobenzene derivatives. Different azobenzene derivatives were developed with different linkers and base analogs and incorporated to different segments of oligonucleotides.

To enatioselectively introduce AzoB into DNA or RNA, optically pure d-threoninol and l-threoninol linkers were employed. d-Threoninol-linked AzoB (D-tAzo)^133^ (Fig. 17A) induces larger changes in *T*_m_ between the *trans* and *cis* isomers than l-threoninol (L-tAzo) (Fig. 17B) and is now commercially available, making it one of the most commonly used forms of AzoB in DNA/RNA.^12^

Introducing methylthio-modification at *para*-position of azobenzene induced a bathochromic (red-) shift of absorption maximum, allowing *trans*-to-*cis* isomerization by 400 nm visible light (Fig. 17C). Additional methylation at the *ortho*-position of the distal benzene ring enhanced the stacking interaction of *trans*-azobenzene while further destabilizing *cis*-AzoB (Fig. 17C). This in turn raised the *T*_m_ of the *trans*-form and lowered the *T*_m_ of the *cis*-form, and the resulting large Δ*T*_m_ enhanced photoregulatory efficiency. More recently, an AzoB modified with a highly branched secondary alkylthio group was incorporated into DNA *via* an l-threoninol scaffold for which the photoisomerization was carried out by visible light (*λ* = 400 nm for the *trans*-to-*cis* reaction with 58% efficiency and *λ* = 520 nm for the reverse reaction) (Fig. 17C). In contrast to other AzoB, these modifications also showed that the *trans*-form is duplex-destabilizing than the *cis*-form (Fig. 17C).^13^

**Fig. 18:**
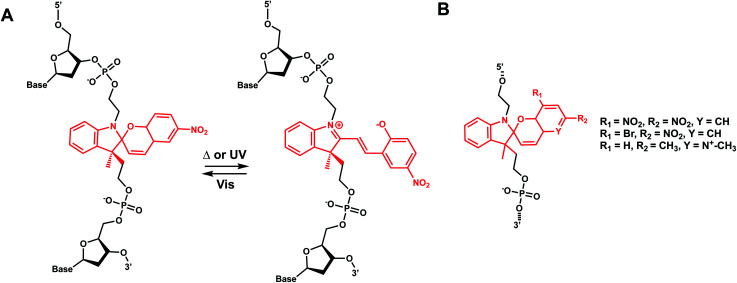
Spiropyran modification on oligonucleotides. (A) Spiropyran-derived DNA backbone linker can undergo reversible isomerization through ring opening/closing with light or heat. (B) DNA modifications synthesized by the Heckel group with different photophysical and thermodynamic properties.

More recently, several modifications have been made to improve the modest photoisomerization efficiency of d-tAzo (*e.g.*, 30% of *cis*-isomer at 37 °C with irradiation with 365 nm (ref. 134)) or l-tAzo.^13^ For instance, Liang *et al.*, introduced AzoB through R-glycerol linker (R-gAzo) which has improved photoisomerization efficiency to 70–80% at room temperature (Fig. 17D).^135^ Later, Asanuma, Heckel, and co-workers developed p-Azo and m-Azo C-nucleosides photoswitches which exhibited complete photoisomerization at room temperature (Fig. 17E and F).^136^

The AzoB group was also incorporated as a part of the backbone in DNA/RNA: Tang *et al.* introduced 4′-bis(hydroxymethyl)-azobenzene to dumbbell hairpin antisense strand complementary to target RNA at the loop position to reversibly control the stability of the hairpin structure *via* UV or visible light (Fig. 17G).^137^

Desaulniers *et al.*, developed photo-regulatable siRNAs with internal azobenzene derivative spacers (siRNAzos) (Fig. 17H).^138–140^ A related, tetra *ortho*-chlorinated azobenzene-containing siRNAs (Cl-siRNAzos) shifted the *trans* to *cis* conversion wavelength to 660 nm (red-shift) and was applied in cell culture gene inactivation studies (Fig. 17H).^141^

AzoB can also be introduced as a part of the purine ring as 2-phenyldiazenyl-substituted 2′-deoxyadenosine (dA_Azo_) and 2′-deoxyguanosine (dG_Azo_) (Fig. 17I and J).^10^ G_Azo_ has been developed by Ogasawara and used as a photoresponsive 5′-cap of mRNA *in vivo* to control protein expression.^142^

#### Reaction characteristics

The UV–vis spectrum of unsubstituted *trans*-azobenzene shows two absorption maxima: a strong one around 320 nm resulting from the symmetry-allowed π–π* transition and a weaker one around 430 nm indicative of the symmetry forbidden n–π* transition. The absorption at about 320 nm leads to rotation around the N=N bond and the formation of the *cis* isomer. The transition associated with the absorption at 430 nm is related to the *cis* to *trans* isomerization. These properties can be influenced by the substitution of the azobenzene core structure and the choice of solvent.

#### Thermodynamic or structural characteristics

AzoB derivatives in oligonucleotides are shown to be isomerized in seconds to minutes range using mW range of power.^131,133,136,143^ Effect of azobenzenes on duplex stability is reviewed by Feringa.^12^ In general, the intercalation of planar *trans*-AzoB stabilizes the DNA or RNA duplexes whereas *cis*-AzoB destabilizes due to non-planarity caused by steric hindrance (Fig. 16). The destabilization effect of the *cis*-Azo was observed by various research groups and for several different azobenzene nucleoside surrogates.^5,13,131,133,136^ In general, the *T*_m_ differences between the *cis*- and *trans*-forms of AzoB modifications in DNA duplexes are ∼1–5 °C.^130,131,136^ dA_Azo_ and dG_Azo_ decreased the *T*_m_ of 10-bp DNA duplexes by 10–13 °C compared with 16 °C of *m*-Azo.^10^

The impact of AzoB on the thermal stability of the DNA also depends on the stereochemical environment of the group. For instance, the *trans*-form of d-tAzoB is more stable than that of l-tAzoB because d-threoninol prefers a clockwise winding, as does the DNA double helix. *Cis*-form is also more destabilized in d-tAzoB than in l-tAzoB, resulting in a larger *trans*-to-*cis* stability difference (Δ*T*_m_) for d-tAzoB.

#### Biological applications

As extensively reviewed by Feringa^12,144^ and Zhang,^145^ AzoB groups have been used in numerous biological applications: regulating hybridization in nucleic acids,^124,146^ transcription of T7 RNA polymerase,^143^ antisense DNA-mediated gene expression,^147^ RNA digestion by RNase H using modified DNA,^148^ nano-tweezer regulation,^149^ and inhibiting DNA aptamer with thrombin.^150^

Newer applications include siRNAzos^151^ in gene silencing in cells and *in vivo*: siRNAzos use AzoB as internal spacers within the sense strand in HeLa cells.^138^ siRNAzos in the *cis* form would distort the siRNA helix, thus rendering it non-functional, but irradiation with UV light would make it functional and lead to gene silencing.^138^ siRNAzos also has been used in the 3′-end of the sense strand with improved nuclease resistance for gene silencing applications.^139^ Red-shifted Cl-siRNAzos were used in cell culture with reversibility.^141^ Additionally, dG_Azo_ developed by Ogasawara was used as a photoresponsive 5′-cap demonstrating the impact of the distal aromatic ring on the dG_Azo_ in the development of double-headed zebrafish by controlling the expression of squint protein.^142^

### 11. Spiropyran

Hirshberg and Fischer reported the first photochemical reactions and photochromic phenomena of spiropyrans.^152^ Spiropyrans are unique among the broad spectrum of photoswitches, due to the range of stimuli (*e.g.*, temperature, visible light, mechanical forces, and solvent effects) able to induce its reversible isomerization.^153^ Spiropyran consists of orthogonally orientated indoline and chromene moieties, joined by a quaternary carbon atom and thus is largely nonplanar.

Early work on spiropyran-modification on DNA oligonucleotides met with various obstacles including fast hydrolysis of spiropyrans in aqueous buffer solutions and the loss of the photoswitching ability in DNA.^6,154^ This problem was largely alleviated when Heckel *et al.* incorporated spiropyran as a part of the DNA backbone using phosphoramidite chemistry and solid-phase synthesis (Fig. 18).^6^ This photoswitch was also reported to work when incorporated at the 5′-end of homothymidine oligonucleotide in duplex DNA.^153^

**Fig. 19:**
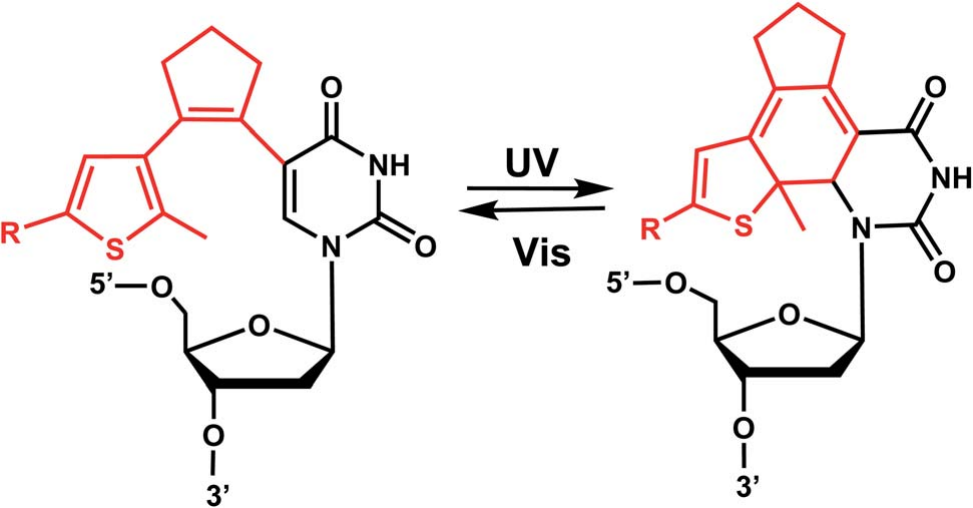
Light-induced reversible [2 + 2] cycloaddition of the diarylethene group. Schematic of the ring formation upon light irradiation at UV range, and reversible reaction ring opening upon light irradiation at visible range.

#### Reaction characteristics

Spiropyran groups incorporated as a linker in the phosphate backbone can undergo heterolytic cleavage of the C_spiro_–O bond either by thermal or photochemical perturbation (*λ*_max_ = 365 nm).^6^ Cleavage of the C_spiro_–O bond leads to the formation of the zwitterionic planar merocyanine due to the extended π-electron system (absorption around 400 nm).^6^ This ring-opening accompanies a large change in dipole moment (Δ*μ* = 7–15 D) and thus increases the overall polarity of the group.^155^ The change is more pronounced than with other reversible photoswitches such as azobenzenes or diarylethenes.^6^ The closed form can be regenerated by thermal energy or upon visible light irradiation (*λ*_max_ = 530 nm).^6^ The equilibrium in the photostationary state can be tuned both by the nature of the substituents or by the solvents used.^12^ Notably, the spectral and photophysical properties of spiropyrans are tunable by changing the substitution pattern in a variety of positions. Different substitutions of spiropyran rings with different photophysical and thermodynamic properties have been reported.^6^

#### Thermodynamic or structural characteristics

There is no melting temperature study for spiropyran included internally as a linker in the phosphate backbone. However, SP added at the end of an 8-bp (dT)_8_:(dA)_8_ duplex showed that merocyanine (open-form) has a lower *T*_m_ by 3–4 °C compared with the spiropyran (closed form).^153^ Another study showed that a non-reversible version of spiropyran modification (using click chemistry) in 17-bp DNA duplexes showed significant destabilization (−12 to −20 °C).^154^

#### Biological applications

Not reported.

### 12. Diarylethene group

Diarylethenes (DAE) are known for excellent photochromic properties, such as negligible thermal relaxation, spectral tunability, and strong absorption bands upon photoconversion as well as high fatigue resistance against multiple photoswitching cycles.^156,157^ Diarylethenes containing thiophene moieties and a cyclopentene ring are a special class of stilbene-type structures in which the *ortho*-hydrogens are substituted to suppress irreversible oxidation after photocyclization of the *cis* isomer. Typically, the incorporated aryl rings are replaced by heterocycles to elongate the lifetime of the closed form, and the ethene moiety is often embedded in a small ring to prohibit *cis*–*trans* isomerization (Fig. 19).

**Fig. 20:**
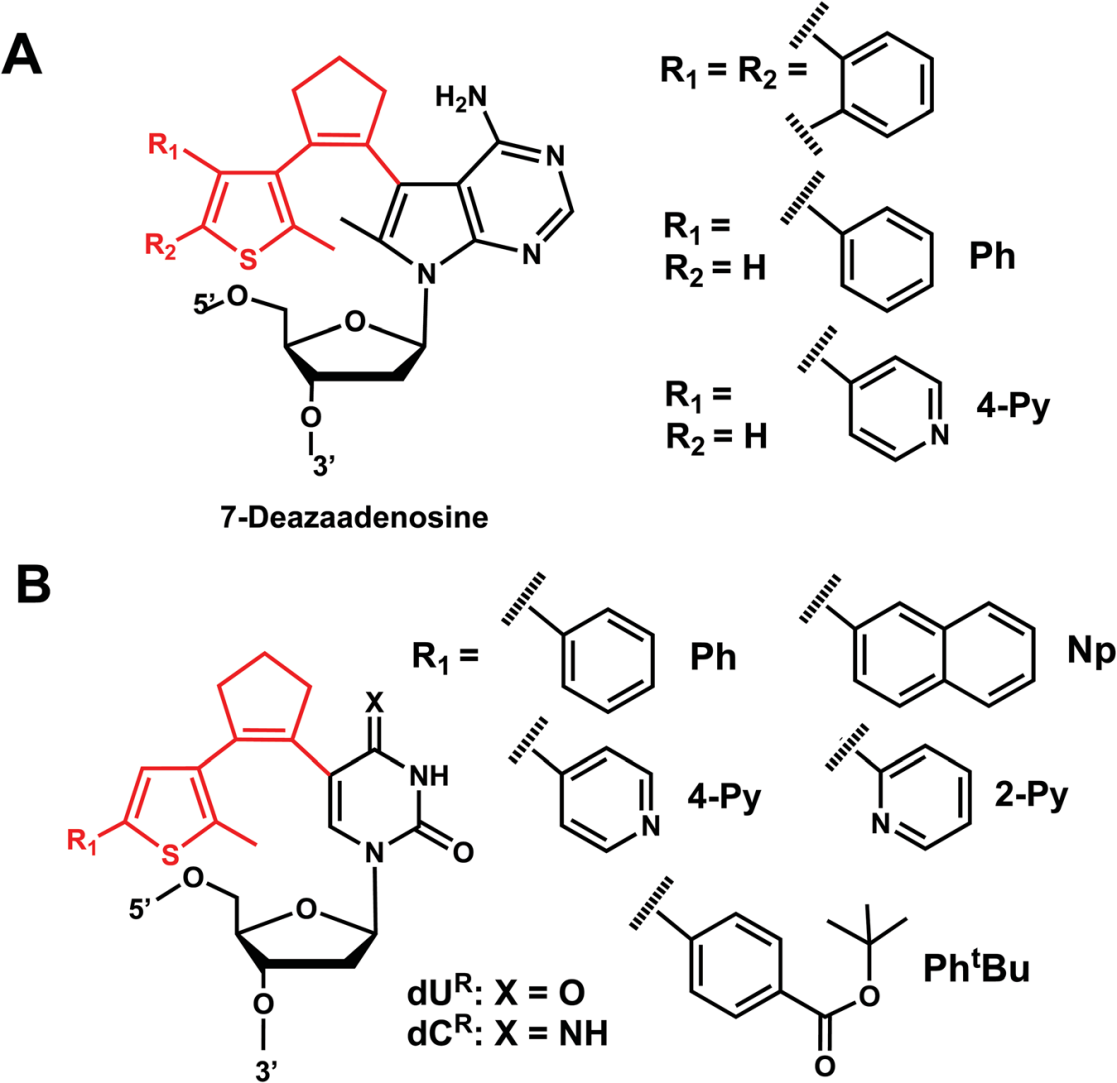
Diarylethene group modification on oligonucleotides. (A) Diarylethene-derived modifications on 7-deazaadenosine nucleoside analogue. (B) Diarylethene-derived modifications on pyrimidine nucleosides.

Diarylethene derivatives were first introduced to oligonucleotides by Jäschke's group through 7-deazaadenosine.^158^ Diarylethene under the irradiation of different wavelengths (250–370 nm) undergoes an electrocyclic rearrangement, generating strongly colored closed-ring isomers, whereas visible light (>400 nm) triggers the cyclo-reversion to the colorless opened-ring form which is thermally stable (Fig. 19).^158^

Originally reported as a photoswitchable reaction in non-aqueous solvent,^158^ the relatively low efficiency of photoisomerization in aqueous solvents had to be optimized using various substituents on the thiophene ring on 7-deazaadenosine which resembles purine^159,160^ or on a (deoxy)uridine as a pyrimidine analog (Fig. 20).^161,162^ In comprehensive testing of 13 different substituents on dU and dC nucleosides, dU with 2-pyridyl (2Py) and *tert*-butylester-phenyl (Ph^*t*^Bu) were found to be the best in the photoisomerization efficiency and thermal and photochemical stability (Fig. 20).^163^ In particular, the photochromism (*e.g.*, quantum yields, composition of the photostationary states, thermal and photochemical stability, and reversibility) of the modified dU with 2-Py or Ph^t^Bu was maintained in the environment of the single-stranded oligonucleotide, and for Ph^t^Bu even in the duplex. These modifications were also shown to be useful in controlling transcriptional activation.^163^

#### Reaction characteristics

The characteristic absorption bands of the diarylethene chromophore at *λ* = 242 nm and *λ* = 305 nm are detectable in the UV/Vis spectrum of the modified nucleoside (colorless solutions-yellow). Irradiation by UV (250–370 nm) in the range of 5–30 min closed the diarylethene moiety and the visible absorption band rose at *λ* = 450 nm (strongly red). Closed isomers of DAE share the emergence of a broad absorption band between 400 and 600 nm with different maxima depending on the thiophene substitution extension of the conjugated system.^158^ Other photophysical and chemical properties, such as isomerization wavelengths, quantum yields, thermal stability, and fatigue resistance can also be tuned by various substituents in the thiophene or cyclopentene ring.^162^ The use of a broad range of light including low energy visible wavelengths is one of the strengths of DAE modification, which can be useful for biological applications.

#### Thermodynamic or structural characteristics

Incorporation of one uridine-caged diarylethene substituted with phenyl group in the thiophene ring decreased the *T*_m_ of the DNA duplex by 2.3 °C in both the open and closed ring forms compared with that of the unmodified duplex.^160^ 3′- or 5′-terminal modifications were found to have a negligible effect on the stability in the open-ring form.^160^ CD spectra of the same DNA, showed an apparent shift to a more A-like (*i.e.*, RNA-like) conformation compared with natural DNA. The spectra were almost identical to unmodified DNA when the modification was terminal, and their UV-induced DNA conformational changes were also small.^160^

#### Biological applications

In the study by Jäschke's group, a single diarylethene modification of dU^R^ with 2-Py, Ph^t^Bu moieties positioned within T7 promoters were shown to modulate transcription rate in *in vitro* transcription assays.^163^ The open-ring form containing promoters showed almost the same activity as the unmodified controls, whereas a ∼2 fold decrease in the transcription rate was observed for the closed form after UV irradiation.^163^

## Concluding remarks

Photoconvertible groups offer a convenient way to alter molecular structures in a spatially and temporally controlled manner using light as the reaction initiator. Ideal photoreactive groups for biological applications would feature fast and complete photoconversions under mild, physiologically relevant conditions and would be capable of multiplexed, orthogonally controllable reactions (*e.g.*, by choosing different wavelengths of trigger light). In recent years, significant strides have been made in the availability and applicability of photoreactive oligonucleotides. Here, we compiled a list of currently available photoreactive groups for oligonucleotides to regulate DNA/RNA structure and function for diverse biological applications (Table 1). The photoreactions are either irreversible (*e.g.*, cleavage) or reversible (*e.g.*, crosslink, isomerization, and intramolecular cyclization reactions), each with their own strengths but also limitations. For instance, reactions that use UV light may cause DNA or tissue damage and can interfere with the excitation/emission of fluorescent reporters used in *in vitro*/*in vivo* studies. Relatively moderate or low photoconversion yields (reaction completeness) of photoreactive groups also remain as hurdles. Expanding the array of available photoreactive modifications with enhanced photostability, biocompatibility and tunability would be exciting future directions.

Continued research and development of light-convertible oligonucleotides promise to provide powerful tools for studying complex genetic mechanisms that uses DNA/RNA as their platforms including transcription, replication, and repair. For example, for DNA repair pathways such as NER where bulky lesions are recognized and repaired, *o*-nitrobenzyl, p-hydroxyphenacyl, and coumarin-related modifications may be used as a bulky DNA lesion surrogate which can be readily switched on and off for various structural and mechanistic studies. Further improvements of photoconvertible nucleic acids to achieve higher photo-reaction efficiency and tunability of light at longer wavelengths may expand the applicability of photoreactions for biological investigations and modulation.

**Table 1:** Photochemical modifications for DNA/RNA oligonucleotides^e^

#	Photoreaction type	Photoreactive group	Structure	Reaction wavelength (nm)	Reversible?^b^	*T*_m_ effect	Photoreaction time scale	Nucleic acid position	Biological application
1	(I) Photocleavage – irreversible	*o*-Nitrobenzyl^a^ (& NPE, NPM, NPOM, PNVOM, NDBF, *etc*)	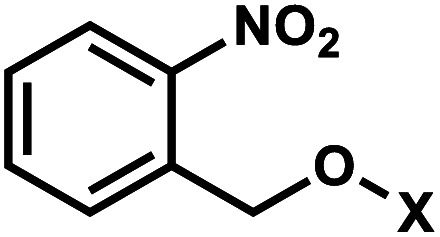	345–420	No	↓	sec–min	Bases, backbone, ribose	Variety of biological systems (see text)
2	(I) Photocleavage – irreversible	*p*-Hydroxyphenacyl (& HBT)	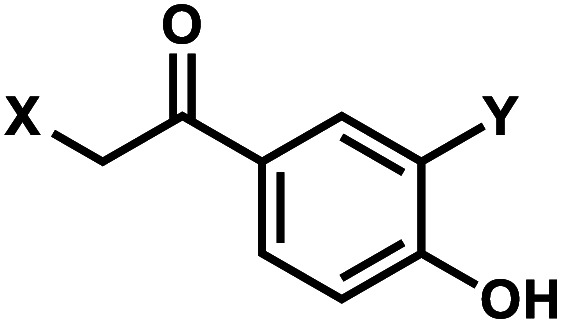	295–405	No	↓	msec–sec	G, T bases	Control of antisense RNA annealing
3	(I) Photocleavage – irreversible	TEEP-OH	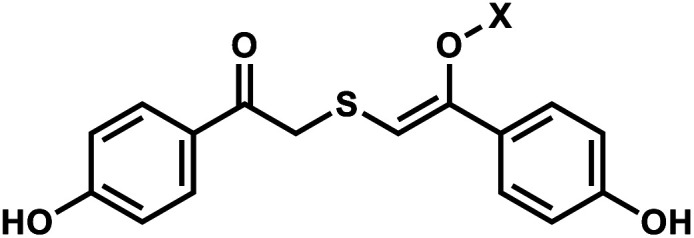	300–365	No	ND	min	Phosphate backbone on DNA	Regulation of DNAzyme activity
4	(I) Photocleavage – irreversible	Aryl sulfide	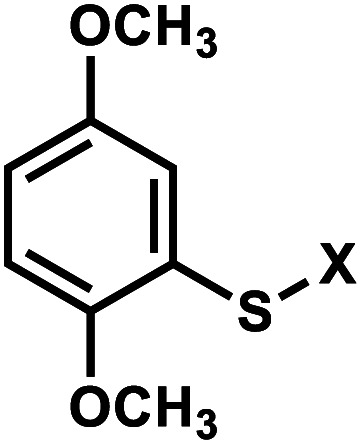	350	No	↓	μsec–min	U, T bases	Control of RNA riboswitch folding
5	(I) Photocleavage – irreversible	Nitroindole	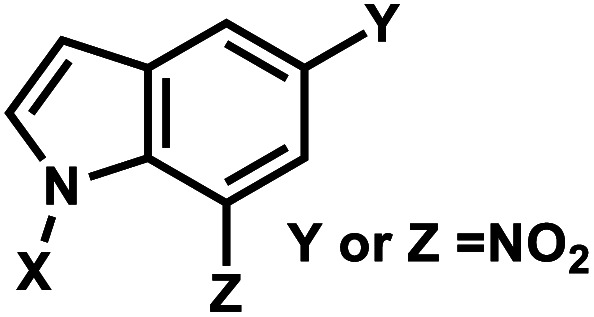	350	No	↓	min	Deoxyribose as a purine analogue	Catch and Release DNA Decoys
6	(I) Photocleavage – irreversible	Benzophenone, acetophenone	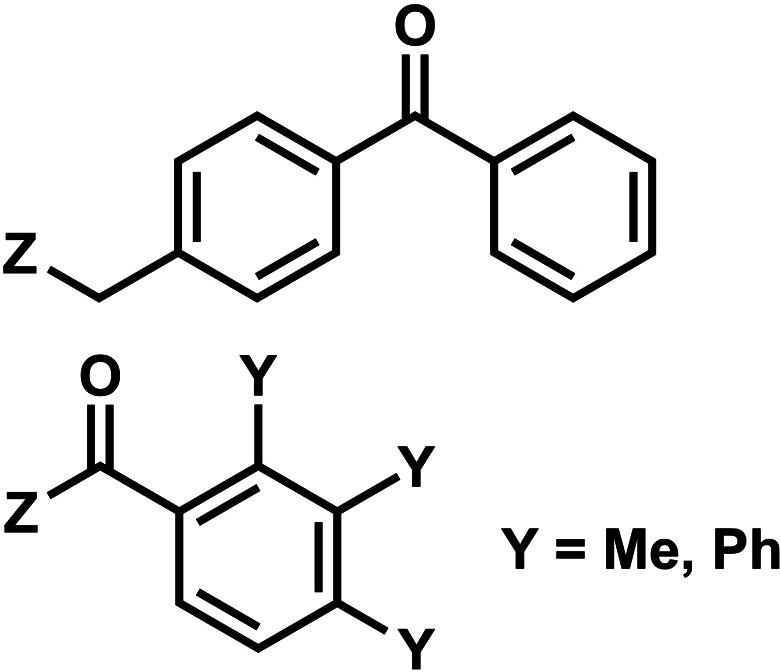	365	No	ND	min	G, C bases	Regulation of mRNA translation; photocrosslink with bound proteins
7	(I) Photocleavage – irreversible, (II) Intermolecular photocrosslinking *via* [2 + 2] cycloaddition – reversible	Coumarin (& DEACM, Bhc; quinoline. *Cf.* psoralen)	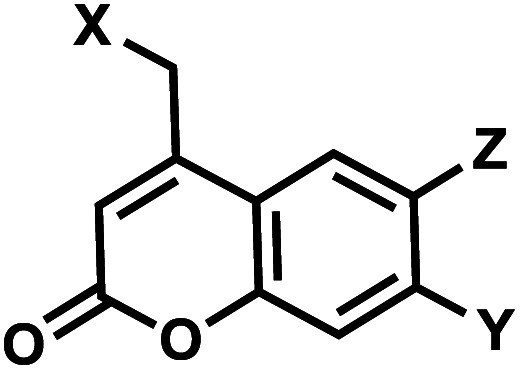	350–470	No for cleavage; Yes for crosslinking (254)	↓	sec–min	G, T bases, phosphate, backbone linker	Catch-and-release DNA decoy, regulation of mRNA caging, transient DNA polymerization, aptamer
8	(II) Intermolecular photocrosslinking *via* [2 + 2] cycloaddition – reversible	Carbazole^a^ (&^CNV^K, ^CNV^D, ^PC^X, and ^PCX^D)	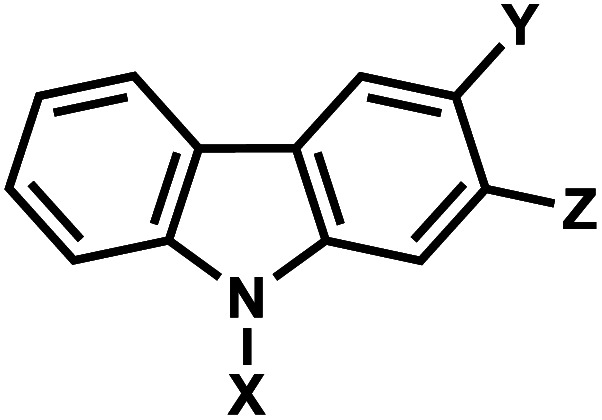	365–450	Yes (312)	↓↑	sec–min	Nucleoside	RNA FISH, plasmid labeling, antisense DNA, regulation of DNAzyme activity
9	(II) Intermolecular photocrosslinking *via* [2 + 2] cycloaddition – reversible, (III). *Cis*–*trans* photoisomerization – reversible	Vinyl-derivative (& stilbazole, cyanostilbene, styrylpyrene; 8ST, 8NV and 8FV)	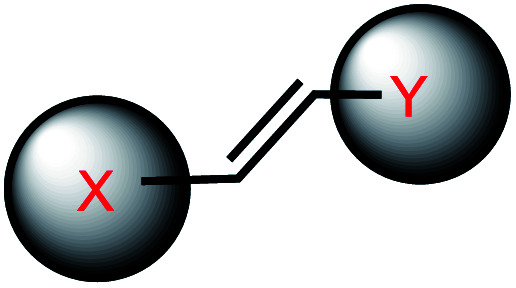	340–465^c^, 370–420^d^	Yes (≤300–340^c^) (254–310^d^)	↓↑	min–hour	Nucleoside, G base	DNA hybridization, regulation of gene expression
10	(III) *Cis*–*trans* photoisomerization – reversible	Azobenzene^a^	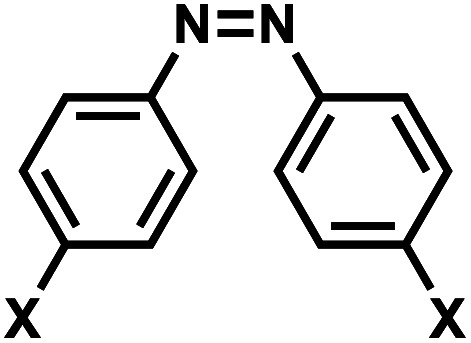	365	Yes (400–420)	↓↑	sec–min	Nucleoside backbone linker, G, A bases	Variety of biological systems (see text)
11	(IV) Intramolecular photocyclization – reversible	Spiropyrans	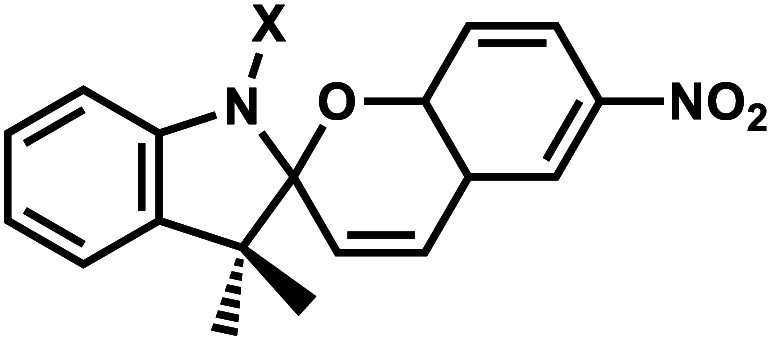	365	Yes (400–520)	ND	min–hour	Backbone	ND
12	(IV) Intramolecular photocyclization – reversible	Diarylethene	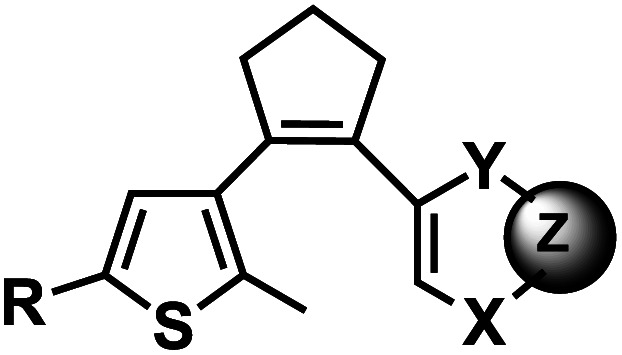	250–370	Yes (>400)	↓	sec–min	U, C bases, A analogue (deazapurine)	ND

a Some derivatives (*e.g.*, d-tAzo) are commercially available.

b Wavelengths in parentheses indicate those for the reverse, uncrosslinking reactions.

c Wavelengths for (un)crosslinking reaction.

d Wavelengths for *cis*–*trans*/*trans*–*cis* isomerization.

e N.B. All modifications can be incorporated in solid-phase oligonucleotide syntheses (*e.g. via* phosphoramidite chemistry) unless otherwise noted (*e.g.*, DMNEC as a part of *o*NB, TEEP-OH, and DPMTC as a part of coumarin).

## Abbreviations

NPE: 1-(*ortho*-Nitrophenyl)-ethyl
NPP: 2-(*ortho*-Nitrophenyl)-propyl
DMNEC: 1-(4-(2-(Dimethylamino)ethoxy)-5-methoxy-2-nitrophenyl)ethyl carbonyl
HBT: 2-(2′-Hydroxyphenyl) benzothiazole
dA_Azo_: 2-Phenyldiazenyl-substituted 2′-deoxyadenosine
dG_Azo_: 2-Phenyldiazenyl-substituted 2′-deoxyguanosine
2Py: 2-Pyridyl
^CNV^K: 3-Cyanovinylcarbazole
^CNV^D: 3-Cyanovinylcarbazole modified d-threoninol
DMNPE: 4,5-Dimethoxy-2-nitrophenylethyl
Bhc: 6-Bromo-7-hydroxycoumarin-4-ylmethyl
NPOM: 6-Nitropiperonyl hydroxymethylene
DEACM: (7-Diethylaminocoumarin-4-yl)methyl
BHQ-diazo: 8-Bromo-2-diazomethyl-7-hydroxyquinolinyl
8FV: 8-Fluorenylvinyl
8NV: 8-Naphthylvinyl
^NV^A: 8-Naphthylvinyladenine
^PV^A: 8-Pyrenylvinyl adenine
8ST: 8-Styryl
ATP: Adenosine triphosphate
ArS: Aryl sulfide
BP: Benzophenone
CRDDs: Catch and release DNA decoys
CRISPR: Clustered regularly interspaced short palindromic repeats
Cas9: CRISPR-associated protein 9
DNA: Deoxyribonucleic acid
DAE: Diarylethenes
DSBs: Double-strand breaks
d-tAzo: d-Threoninol-linked AzoB
^PCX^D: d-Threoninol version of the ^PC^X
FISH: Fluorescence *in situ* hybridization
GFP: Green fluorescent protein
gRNA: Guide RNA
ICL: Interstrand crosslink
d-tAzo: l-Threoninol-linked AzoB
*T*_m_
: Melting temperature
mRNA: Messenger RNA
NDBF: Nitrodibenzofuran
NF-κB: Nuclear factor κB
NMR: Nuclear magnetic resonance
*o*NB: *Ortho*-Nitrobenzyl
PSS: Photostationary state
*p*HP: *p*-Hydroxyphenacyl
PNVOM: Propargyl-6-nitroveratryloxymethyl
^PC^X: Pyranocarbazole
R-gAzo: R-Glycerol-linked AzoB
RNA: Ribonucleic acid
SNA: Serinol nucleic acid
sgRNA: Single guide RNA
siRNA: Small interference RNA
SP: Spiropyran
S_P_: Styrylpyrene
Ph^*t*^Bu: *tert*-Butylester-phenyl
TFO: Triplex-forming oligonucleotides
TLR9: Toll-like receptor 9
TEEP-OH: Thioether-enol phosphate, phenol substituted
UV: Ultraviolet
vfCRISPR: Very fast CRISPR
Vis: Visible

## Conflicts of interest

There are no conflicts to declare.

## Supplementary Material
